# Site-specific glycoproteomic analysis revealing increased core-fucosylation on FOLR1 enhances folate uptake capacity of HCC cells to promote EMT

**DOI:** 10.7150/thno.56882

**Published:** 2021-05-08

**Authors:** Li Jia, Jun Li, Pengfei Li, Didi Liu, Jing Li, Jiechen Shen, Bojing Zhu, Chen Ma, Ting Zhao, Rongxia Lan, Liuyi Dang, Wang Li, Shisheng Sun

**Affiliations:** 1 College of Life Science, Northwest University, Xi'an, Shaanxi province 710069, China.; 2 Key Laboratory of Regenerative Biology, Guangzhou Institutes of Biomedicine and Health, Chinese Academy of Sciences, Guangzhou, Guangdong province 510530, China.

**Keywords:** hepatocellular carcinoma, epithelial-mesenchymal transition, glycoproteome, FOLR1, core-fucosylation

## Abstract

**Rationale:** Epithelial-mesenchymal transition (EMT) has been recognized as an important step toward high invasion and metastasis of many cancers including hepatocellular carcinoma (HCC), while the mechanism for EMT promotion is still ambiguous.

**Methods:** The dynamic alterations of site-specific glycosylation during HGF/TGF-β1-induced EMT process of three HCC cell lines were systematically investigated using precision glycoproteomic methods. The possible roles of EMT-related glycoproteins and site-specific glycans were further confirmed by various molecular biological approaches.

**Results:** Using mass spectrometry-based glycoproteomic methods, we totally identified 2306 unique intact glycopeptides from SMMC-7721 and HepG2 cell lines, and found that core-fucosylated glycans were accounted for the largest proportion of complex *N*-glycans. Through quantification analysis of intact glycopeptides, we found that the majority of core-fucosylated intact glycopeptides from folate receptor α (FOLR1) were up-regulated in the three HGF-treated cell lines. Similarly, core-fucosylation of FOLR1 were up-regulated in SMMC-7721 and Hep3B cells with TGF-β1 treatment. Using molecular approaches, we further demonstrated that FUT8 was a driver for HGF/TGF-β1-induced EMT. The silencing of FUT8 reduced core-fucosylation and partially blocked the progress of HGF-induced EMT. Finally, we confirmed that the level of core-fucosylation on FOLR1 especially at the glycosite Asn-201 positively regulated the cellular uptake capacity of folates, and enhanced uptake of folates could promote the EMT of HCC cells.

**Conclusions:** Based on the results, we proposed a potential pathway for HGF or TGF-β1-induced EMT of HCC cells: HGF or TGF-β1 treatment of HCC cells can increase the expression of glycosyltransferase FUT8 to up-regulate the core-fucosylation of *N*-glycans on glycoproteins including the FOLR1; core-fucosylation on FOLR1 can then enhance the folate uptake capacity to finally promote the EMT progress of HCC cells.

## Introduction

Hepatocellular carcinoma (HCC), which ranks sixth among the most common malignancies and fourth in mortality among all kinds of cancers in the world, causes about 841,000 new cases and 782,000 deaths annually 1. Although improved early diagnosis and surgical treatment in recent years have led to elevated survival of patients with HCC, the 5-year survival rate is still less than 20% 2. The main cause of such poor prognosis and high mortality is the recurrence and metastasis of HCC 3, 4.

Epithelial-mesenchymal transition (EMT), which is mainly involved in the transient de-differentiation of epithelial cells into mesenchymal phenotypes, has been recognized as a major trigger for cancer metastasis and invasion in recent years 5-8. According to previous studies, EMT is involved in the metastasis of HCC 9, melanoma 10, breast cancer 11, and esophageal cancer 12. With the help from genomics and proteomics tools, many EMT-related transcription factors, genes, miRNAs and proteins have been reported 6, 13-15, which provide valuable data for us to truly understand the intrinsic changes with EMT. In spite of that, the detailed molecular mechanism for EMT is still ambiguous.

Glycosylation is one of the most extensive and important protein post-translational modifications and participates in many essential biological processes including the cell growth, molecular recognition, signal transduction, immune defense, and other biological processes in normal tissue. The alteration of glycosylation has been proved to be associated with the progression and metastasis of many cancers, such as bladder 16 and breast cancers 17. Especially, the abnormality of *N*-glycan on the cell surface has been proved to be associated with the metastasis and EMT of HCC 18, 19. One example of EMT-related glycan changes is that the increased core-fucosylation on *N*-glycans catalyzed by the glycosyltransferase FUT8 20. These studies, however, mainly focus on the analysis of released glycans by using glycomics or lectin microarray approaches 16-19, which are unable to link glycans with their related glycoproteins 19. Therefore, the related functional glycoproteins and detailed mechanism are still largely unknown. In recent years, site-specific glycosylation analysis has been achieved by directly analyzing intact glycopeptides using mass spectrometry 21-25, by which both glycan alterations and their attached glycoprotein information can be clearly profiled. We reason that this glycoproteomic technology has great chance to recover the possible connections between altered glycans and glycosylated proteins associated with EMT of cancer cells, and even identify some new site-specific glycan changes related to EMT to facilitate a better understanding of the EMT mechanism.

**Figure 1:**
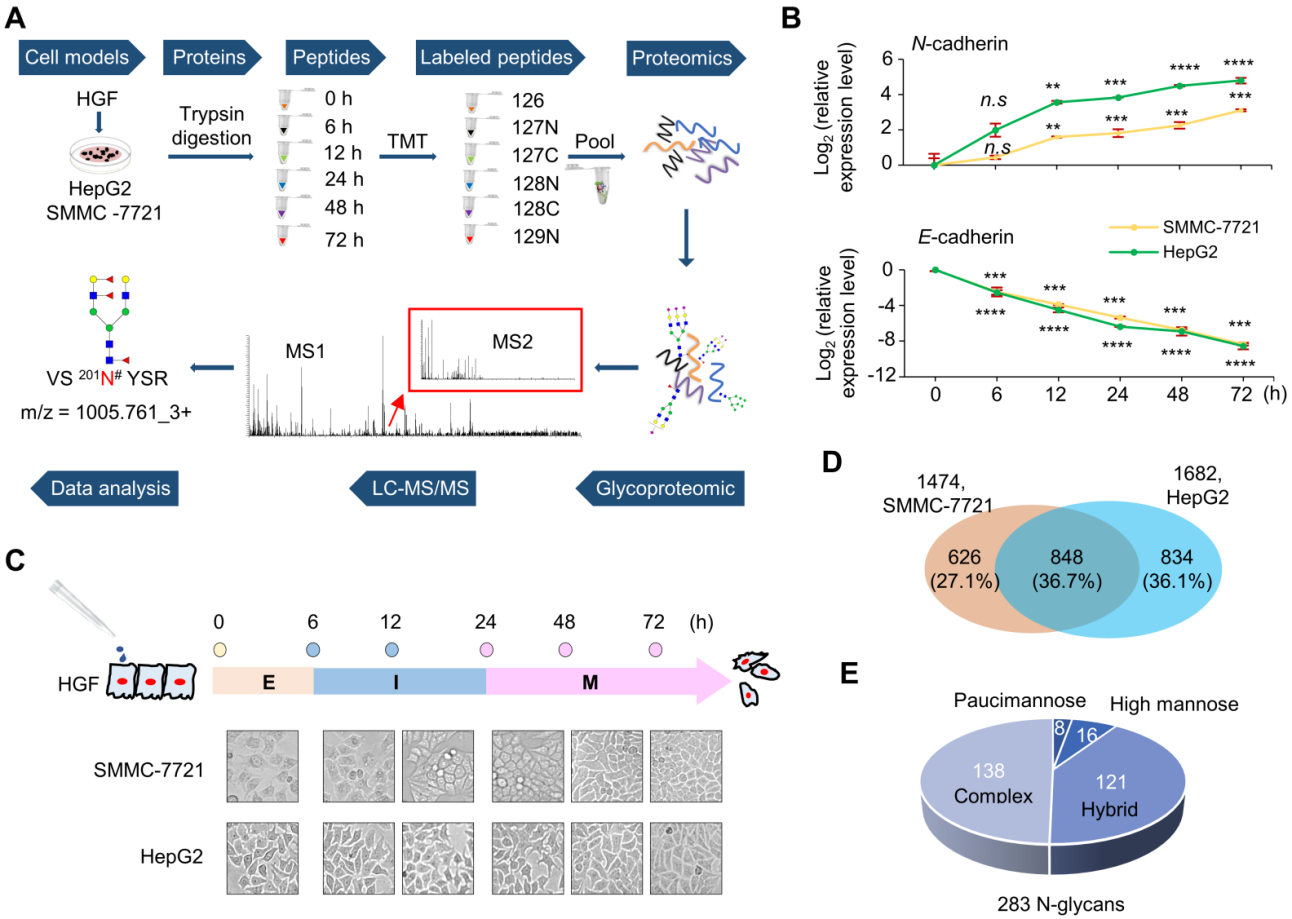
** Epithelial-mesenchymal transition (EMT) of two hepatocellular carcinoma cell lines induced by HGF treatment. A.** Workflow of dynamically site-specific glycosylation and global proteome analyses of two hepatocellular carcinoma cell lines during the EMT process. **B.** The down-regulation of E-cadherin and the up-regulation of N-cadherin were monitored at six time points of HGF treatment (0-72 h) by quantitative real-time PCR (qRT-PCR). Data are presented as mean±SEM of at least triplicates experiments. P values were determined by One-way ANOVA. n.s, no significant; *P < 0.05, **P < 0.01, ***P < 0.001, ****P < 0.0001. **C.** The EMT process could be classified into three stages based on changes of these two EMT markers. E: epithelial stage (0 h), I: intermediate stage (6 h-12 h), M: mesenchymal stage (24 h-72 h). **D.** Comparison of intact glycopeptides identified from SMMC-7721 and HepG2 cell lines. **E.** Distribution of glycan subtypes from intact glycopeptides identified from both cell lines.

In this study, site-specific glycosylation alterations in the EMT process of HCC were systematically investigated using glycoproteomic methods (**Figure 1A**), and the possible roles of EMT-related glycoproteins and site-specific glycans were further investigated by molecular biological approaches. Using these approaches, we revealed that the cellular uptake capacity of folates can be enhanced by increased core-fucosylation on FOLR1 especially at the glycosite Asn-201 to promote the EMT of HCC, which demonstrated a specific role of protein glycosylation in the EMT progress of HCC.

## Results

### HGF triggers EMT-like changes in two hepatocellular carcinoma cell lines

In order to systematically investigate the alterations of site-specific glycosylation in the EMT process of HCC, two widely used HCC cell lines SMMC-7721 and HepG2 were treated by HGF to induce their EMT 26-28, and their whole cellular proteins were harvested at six time points after HGF-treatment (0, 6, 12, 24, 48, and 72 h). Then, the trypsin digested peptides from whole cellular proteins of different time points were labeled by TMT reagents and pooled into one sample for global proteomic analysis with LC-MS/MS. The intact glycopeptides were enriched from pooled samples and analyzed by LC-MS/MS for intact glycopeptide identification and quantification (**Figure 1A**).

The HGF-induced EMT was first evaluated by expression levels of the epithelial marker E‑cadherin and mesenchymal marker N‑cadherin 29. With 10 ng/mL HGF treatment, the mRNA level of E-cadherin decreased to less than 25% within 6 h, and to < 1% after 24 h in both cell lines. The mRNA level of N-cadherin significantly increased within 12 h, and up to 9-fold and 25-fold after 72 h treatment in SMMC-7721 and HepG2 cells, respectively (**Figure 1B**). Based on the expression of EMT markers, the whole EMT process could be divided into three major stages, including epithelial (E: 0 h), intermediate (I: 6 h and 12 h), and mesenchymal stages (M: 24 h, 48 h, and 72 h) (**Figure 1C**). These results demonstrated that HGF treatment caused up-regulated expression of N-cadherin and down-regulated expression of E‑cadherin in a time-dependent manner in both cell lines, indicating the successful induction of EMT by HGF on both cell lines within three days.

The HGF-induced EMT was also confirmed by the relatively high cell growth rates of both HGF-treated cell lines in FBS-free medium, a condition that the untreated cells could not survive. The number of both treated cells increased from 30% to approximately 90% within three days** (Figure S1)**, while the untreated cells decreased from 30% to almost 0% during the same time period (data not shown).

### Site-specific glycosylation profiling in two HCC cell lines

From SMMC-7721 and HepG2 cell lines, we were able to identify 1474 and 1682 unique *N*-linked intact glycopeptides (still with *N*-glycan attached) within 1% false discover rate (FDR), respectively** (Figure 1D** and**Table S1)**. In total, 2306 unique intact glycopeptides, including 848 in common (36.7%), were identified in these two cell lines within 1% FDR (**Figure 1D**). These intact glycopeptides represented 891 glycosites from 657 glycoproteins, modified with 283 glycan structures (159 compositions) **(Figure S2)**. Up to 80 glycans were identified at the glycosite Asn-69 (NACCST^69^N^#^TSQEAHK) of folate receptor alpha (FOLR1). The 283 *N*-glycans contained 16 high mannose, 121 hybrid, 138 complex, and eight paucimannose subtypes of glycans (**Figure 1E**). Three pairs of representative MS/MS spectra, each containing a spectrum from 20% HCD fragmentation and a spectrum from 37% HCD fragmentation, were provided to demonstrate the principle of glycan type determination in the intact glycopeptide analysis (**Figure S3**-**S5**).

### Quantitative intact glycopeptide analysis revealing increased core-fucosylation with EMT

We first performed quantitative proteomic analysis during the EMT process of both cell lines. The relative abundance of each protein was calculated as the ratio of protein expression at the I or M stages versus that of E stage. Among all protein groups identified from SMMC-7721 (2,403 proteins) and HepG2 (1,964 proteins) cell lines, over 99.7% of them were changed within two folds in the I and M stages (**Figure S6A** and**Table S2**). Even though a 1.5-fold change was used as the cutoff, only six increased proteins were identified in the I or M stage compared with the initial E stage in both cell lines (**Figure S6B**). Although more in-depth proteomic analysis may lead to larger numbers of changed proteins identified, the present results clearly indicated that the majority of proteins remained unchanged during the EMT process of both HCC cell lines. Gene ontology (GO) analysis indicated all six proteins were involved in lipids and cholesterol biosynthetic process, in which five of these proteins could interact with each other (**Figure S6C**-**D**). These results suggested that increased synthesis of lipids and cholesterol might be involved in the EMT process induced by HGF, which is consistent with the simulative role of lipid and cholesterol to EMT in prostate cancer 30, breast cancer 31 and ovarian cancer 32. This also agrees with a previous study showing that lipids and cholesterol were harmful metabolites to the liver and their up-regulation could promote the development and growth of HCC 33.

**Figure 2:**
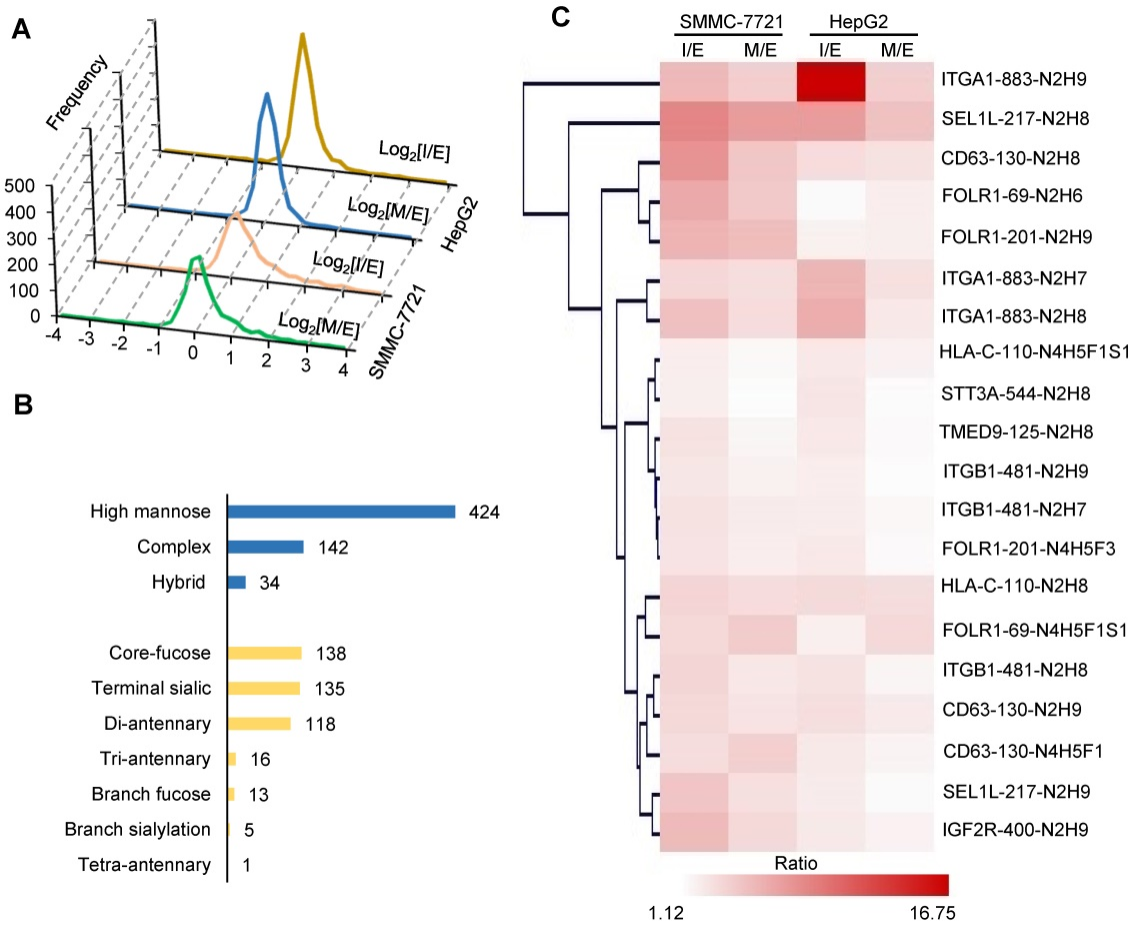
**Quantitative glycoproteomic analysis of two hepatocellular carcinoma cell lines during HGF-induced EMT. A.** Distribution of intact glycopeptide alterations at the intermediate (I) and mesenchymal (M) stages of EMT in both cell lines. *X axis:* log_2_(I/E ratio) or log_2_(M/E ratio); *Y axis:* numbers of intact glycopeptides. **B.** Classification of quantified intact glycopeptides in both cell lines based on their attached glycan structures. The numbers indicate the unique glycopeptides modified by the corresponding glycans. **C.** Hierarchical clustering of altered intact glycopeptides (related to Table S4). The label at the right side: protein name-glycosylation site-glycan composition. For example, ITGA1-883-N2H9 means the protein ITGA1 is modified by the glycan N2H9 at the glycosylation site Asn-883. N: HexNAc; H: Hex; F: Fucose; S: Sialic acid. For example, the glycan “N2H9” indicates a high-mannose glycan that contains two HexNAc and nine Hexoses (also known as Man9).

We then analyzed the quantitative glycoproteomic data to investigate whether glycosylation was changed at the intact glycopeptide level. The distribution of log_2_(I/E) and log_2_(M/E) ratios revealed significant alterations of glycopeptides with approximately 14% and 9% glycopeptides changed more than two folds in the I and M stages of SMMC-7721 cell line, respectively (**Table S3**). Compared with SMMC-7721, fewer glycopeptides were altered (3% and 2% for I and M stages, respectively) in HepG2 cell line (**Figure 2A**). Overall, these results demonstrated a more significant change at glycosylation level than that of protein level during EMT.

**Figure 3:**
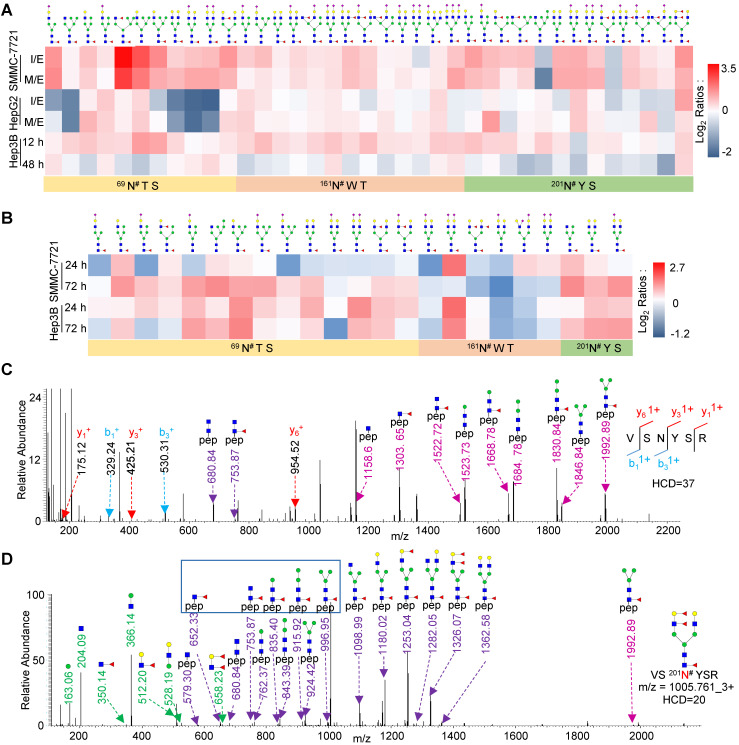
** Site-specific glycan profiling of highly core-fucosylated FOLR1. A.** Heat map showing identified glycans at the glycosite Asn-69, Asn-161, Asn-201 of FOLR1 in three cell lines with HGF treatment. **B.** Heat map showing identified intact glycopeptides from FOLR1 in SMMC-7721 and Hep3B cells with TGF-β1 treatment. **C, D.** Representative MS/MS spectra for identification of an intact glycopeptide from FOLR1. **C.** Identification of the peptide sequence VS^201^N^#^YSR using a MS/MS spectrum with high energy HCD fragmentation. ^#^indicates the glycosylation site. **D.** Determination of the glycan structure HexNAc4Hex5Fuc3 attached at the glycosite Asn-201 using a MS/MS spectrum with low energy HCD fragmentation. Core-fucosylation was identified by five feature Y ions (from peptide+HexNAc_1_Fuc_1_ ion at m/z=652.33^2+^ to peptide+HexNAc_2_Hex_3_Fuc_1_ ion at m/z=996.95^2+^). The outer arm fucosylation was determined based on the feature B ions (HexNAc_1_Hex_1_Fuc_2_, HexNAc_1_Hex_1_Fuc_1,_ and HexNAc_1_Fuc_1_ but no HexNAc_1_Fuc_2_) as well as the related Y ions. The m/z values of Y ions with charge states 1+ and 2+ are labeled by light and dark purple, respectively.

In order to quantify the alterations of intact glycopeptides, we selected 607 unique glycopeptides detected in both cell lines with PSMs ≥ 5. Among them, glycopeptides modified by high mannose glycans accounted for the largest proportion, followed by complex and hybrid glycans (**Figure 2B**). We further analyzed structures of complex glycans using our in-house StrucGP software and found that core-fucosylated glycans accounted for the largest proportion of *N*-glycans, followed by glycans with terminal sialic acids and di-antennary glycans (**Figure 2B**). Quantification results showed that 20 intact glycopeptides from 9 proteins had more than two-fold increases at I or M stage in both cell lines (**Figure 2C**). Among them, four intact glycopeptides were modified with core-fucosylation, including HLA-C modified by N4H5F1S1 at the glycosite Asn-110, CD63 modified by N4H5F1 at the glycosite Asn-130, and FOLR1 modified by N4H5F1S1 and N4H5F3 at the glycosites Asn-69 and Asn-201, and all of them were elevated more than 2-fold at the I and/or M stages in both cell lines **(Figure 2C** and**Table S4)**. Notably, two of them were identified from FOLR1. These results implied that the site-specific core-fucosylation on FOLR1 might play an important role in HGF-stimulated EMT process in HCC cell lines. In addition, these four altered glycopeptides were manually inspected to ensure the correctness of their glycan structure identifications (**Figure 3C-D, S7-9**).

### Core-fucosylation of FOLR1 is upregulated in HCC cells during EMT process

To examine the relationship between the site-specific glycosylation on FOLR1 and HGF-stimulated EMT process, we mapped *N*-linked glycans on each glycosite of FOLR1 based on our glycoproteomics data. A total of 51 unique intact glycopeptides were identified from FOLR1, which were comprised of three glycosites (^69^N^#^TS, ^161^N^#^WT, and ^201^N^#^YS) and 31 glycans (**Table S5A**). Among these glycopeptides, one was modified with paucimannose glycan, 13 were modified with high-mannose glycans, and the other 37 were modified with 23 hybrid or complex glycans (**Figure 3A**). We interestingly found that core-fucosylation was accounted for 62.7% of all glycans on identified intact glycopeptides, including 7 glycans with both core- and branch-fucosylation. The majority of core-fucosylated intact glycopeptides were up-regulated during EMT in SMMC-7721 cell line, and many were also increased in HepG2 cell line (**Figure 3A** and**Table S5A**). In addition, many core-fucosylated intact glycopeptides of FOLR1 were also up-regulated in HGF-stimulated Hep3B cells (**Figure 3A** and**Table S5B**).

We further measured whether core-fucosylation on FOLR1 was also up-regulated in TGF-β1-treated HCC cells. Three cell lines were also treated with 10 ng/mL TGF-β1, and their whole cellular proteins were harvested at three time points (0, 24 and 72 h). Western blot analysis showed that the E-cadherin protein level was decreased, while the N-cadherin protein level was significantly increased in all three TGF-β1-treated cell lines, indicating the successfully induction of EMT by TGF-β1 (**Figure S10**). After LC-MS/MS analysis, we found that most of the core-fucosylation of FOLR1 were also up-regulated in TGF-β1-treated SMMC-7721 and Hep3B cells, especially the glycosylation of glycosite Asn-201 (**Figure 3B** and**Table S6**), which was consistent with the results of HGF treatment. In fact, we also harvested and analyzed TGF-β1-treated HepG2 cells by LC-MS/MS. Unfortunately, the intact glycopeptides from FOLR1 were not identified, which most likely was due to the low expression level of FOLR1 in HepG2 cells (**Figure S10**).

A representative pair of spectra from an up-regulated glycopeptide modified by the glycan HexNAc_4_Hex_5_Fuc_3_ is shown in **Figure 3C**-**D**. In the spectra, the peptide sequence VS^201^N^#^YSR was identified based on the b/y ions of the intact glycopeptide fragmented by the high HCD energy (**Figure 3C**), while the glycan structure was determined based on the B/Y ions of the intact glycopeptide fragmented by the low HCD energy (**Figure 3D**). These results indicated that FOLR1 is a highly core-fucosylated glycoprotein and many core-fucosylated glycans were increased with the EMT in both HCC cell lines, further proved that core-fucosylation of FOLR1 might be involved in the EMT progress of HCC cells.

### Core-fucosylation is mainly regulated by FUT8 during EMT

**Figure 4:**
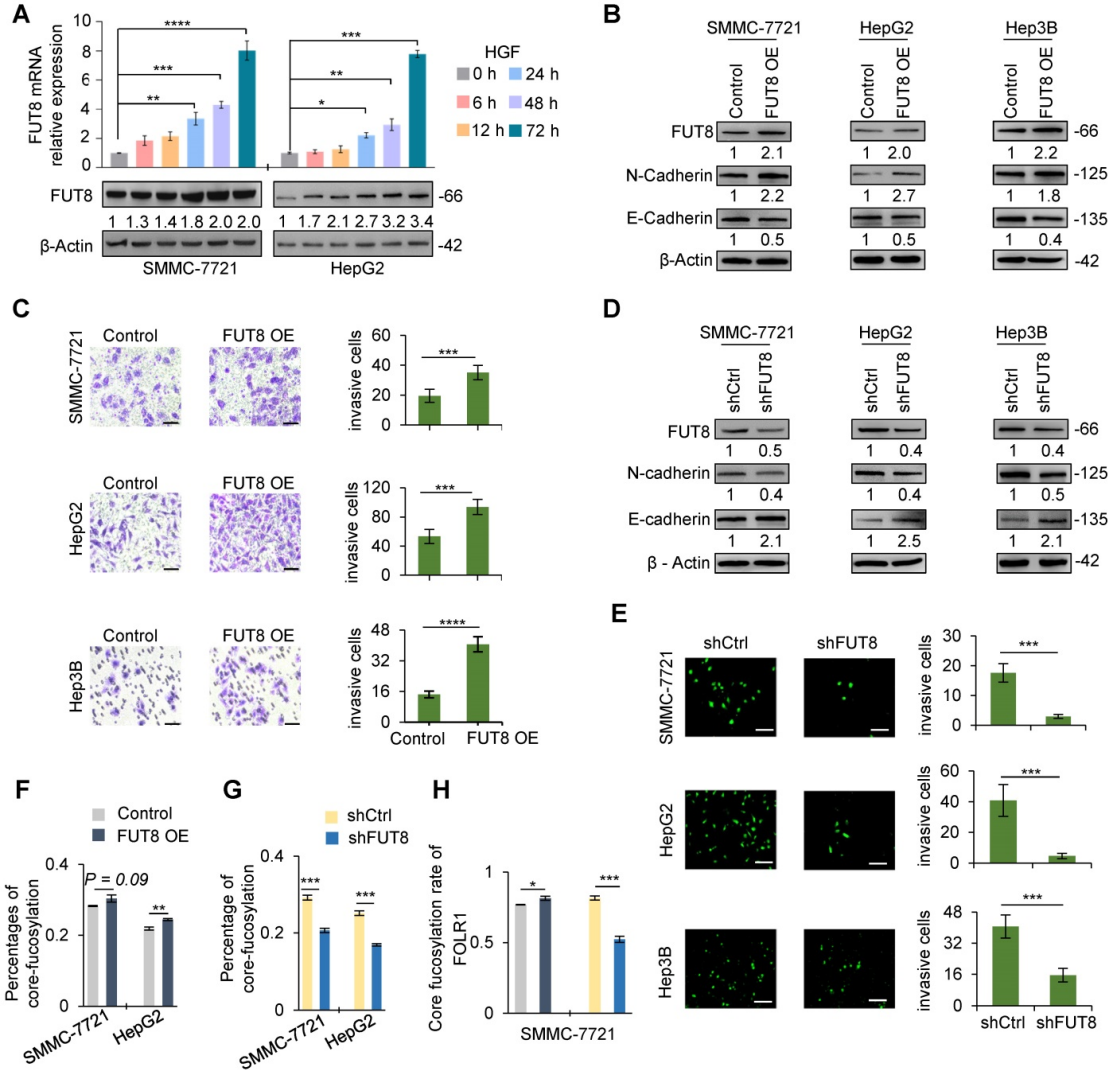
**FUT8 promoted invasive ability of three HCC cell lines. A.** The mRNA (upper) and protein (lower) levels of FUT8 in HGF-treated SMCC-7721 and HepG2 cell lines. **B.** The protein levels of FUT8, N-cadherin and E-cadherin in three stable cell lines with FUT8 overexpression. The grayscale values (lower) were measured from the western blotting data with Image J. **C.** The Matrigel Trans-well invasion assay (Scale bar = 10 µm) in stable HCC cell lines of FUT8 overexpression (FUT8 OE). **D.** The protein levels of FUT8, N-cadherin and E-cadherin in three pairs stable cell lines with FUT8 knockdown. **E.** The Matrigel Trans-well invasion assay (Scale bar = 50 µm) in stable HCC cell lines with FUT8 knockdown (shFUT8). In Matrigel Trans-well invasion assay, invading cells were measured by counting the numbers of cells that were invaded into the basal side of Matrigel-coated trans-well inserts after 24 h incubation (3 replicates per condition, 5 fields per replicate). **F, G.** Percentages of core-fucosylated glycopeptides among all identified glycopeptides based on their spectra counts in two stable HCC cell lines of FUT8 overexpression (**F**) and knockdown (**G**). **H.** The percentage of core-fucosylated glycans on FOLR1 in SMMC-7721 cells. Intact glycopeptide analyses were performed in triplicates for each condition. Data are presented as mean±SEM; P-values were determined by One-way ANOVA (A) or unpaired two-tailed t-test (C, E, and F-G). *P < 0.05, **P < 0.01, ***P < 0.001, ****P < 0.0001.

It is well-known that FUT8 is the sole enzyme to generate a-1, 6-fucosylated structures (core-fucosylation) on *N-*glycans 34. To investigate the role of core-fucosylation in the process of HGF or TGF-β1-induced EMT, we first detected the expression of FUT8 at both mRNA and protein expression levels in HGF-treated cells using qRT-PCR and western blot, respectively. The results indicated that FUT8 was significantly increased at both mRNA and protein levels within 24 h of HGF treatment. Especially, the mRNA levels of FUT8 were increased up to 8-fold after 72 h treatment in both SMMC-7721 and HepG2 cells (**Figure 4A**). Similarly, the expression levels of FUT8 protein were significantly up-regulated in three TGF-β1-treated cell lines (**Figure S10**). These results indicated that FUT8 was positively correlated with the increase level of core-fucosylated glycans with EMT.

To demonstrate the relationship between FUT8 and EMT, we further over-expressed FUT8 in three cell lines in the absence of HGF and TGF-β1 treatment (**Figure 4B, S11A**). As shown in **Figure 4B, S11B**, overexpression of FUT8 significantly increased the protein and mRNA expression level of N-cadherin, which is a key marker of EMT, while significantly decreased the protein and mRNA expression of E-cadherin, indicating that up-regulation of FUT8 could trigger the EMT of HCC cell lines, a similar effect as the HGF or TGF-β1 treatment. We next investigated whether aberrant FUT8 expression promoted the migration and invasion of three cell lines using trans-well invasion assay and crystal violet stain. As expected, overexpression of FUT8 consistently increased the invasive ability of three cell lines (**Figure 4C**).

To further consolidate the above observations, we silenced endogenous FUT8 in three cell lines by short hairpin RNAs targeting FUT8 (shFUT8) (**Figure 4D, S11C**). Western blot and qRT-PCR results showed that knockdown of FUT8 could cause a significant increase of E-cadherin and a significant decrease of the N-cadherin in stable cell lines in the absence or presence of HGF treatment (**Figure 4D, S11D**), revealing that the EMT of three cell lines induced by HGF could be relieved by knockdown of FUT8. To further assess the role of core-fucosylation in migration and invasion of HCC cells, we established stable cell lines of FUT8 knockdown in three cell lines. Then, the effect of FUT8 knockdown on migration and invasion of HCC cells were determined in three cell lines through matrigel trans-well invasion assay. Consistent with our observations, the invasive ability of the stable cell line of FUT8 knockdown was significantly reduced (**Figure 4E**). In addition, the mass spectrometry data showed that overall core-fucosylation were significantly increased in FUT8 overexpressed SMMC-7721 and HepG2 cells (**Figure 4F**), and decreased in FUT8 knockdown cell lines (**Figure 4G**). And the core-fucosylation rate of FOLR1 were also significantly increased with FUT8 over-expression, and decreased with FUT8 knockdown in SMMC-7721 cells (**Figure 4H**). These results provided strongly evidence that increased core-fucosylation is mainly regulated by FUT8 during EMT of HCC cell lines.

### Core-fucosylation enhances the folate uptake capacity of FOLR1

**Figure 5:**
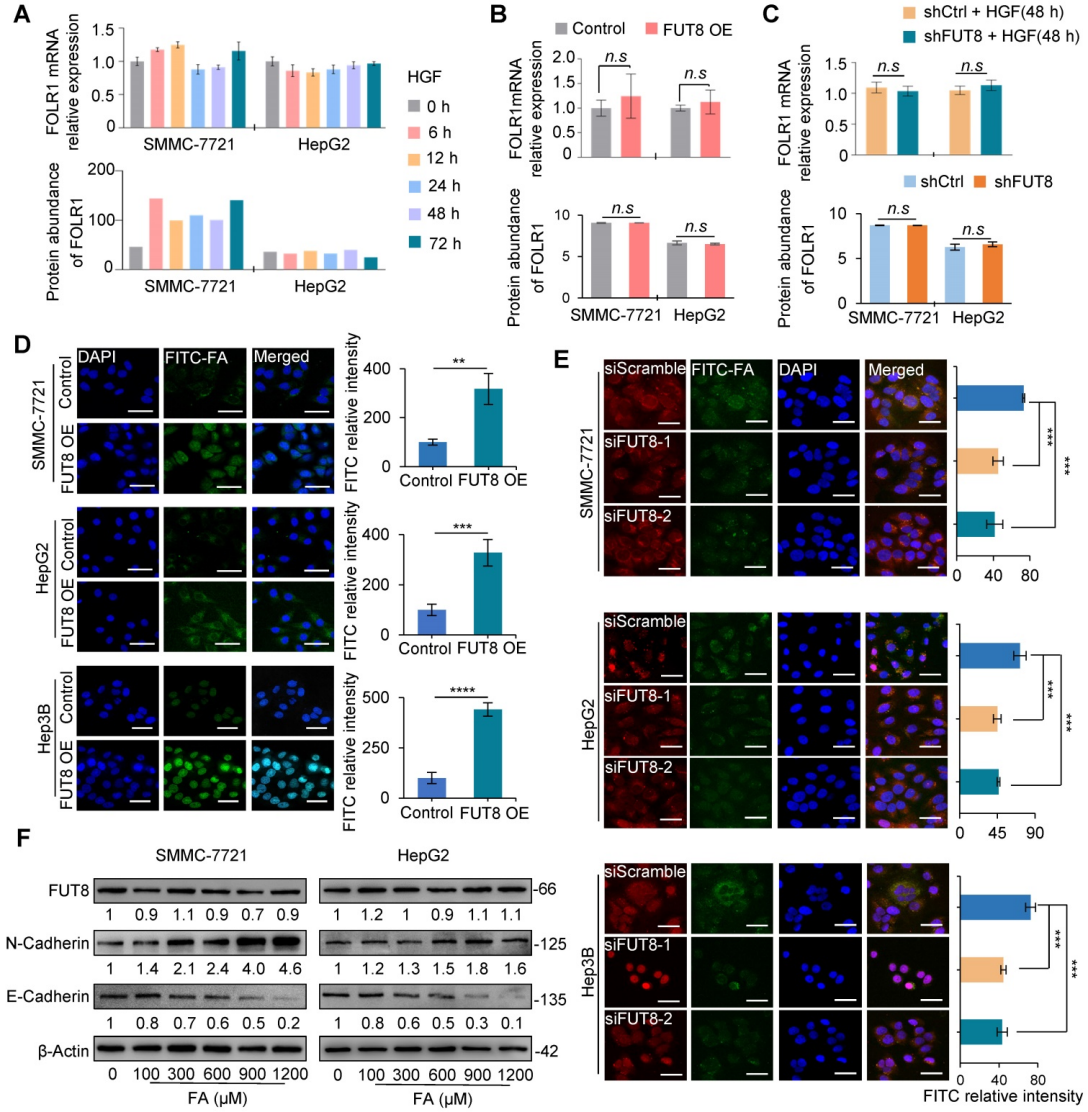
**Core-fucosylation on FOLR1 increased uptake capacity of folate. A.** The mRNA (upper) and protein (lower) levels of FOLR1 in SMMC-7721 and HepG2 cell lines treated by 10 ng/mL HGF for 72 h. **B, C.** The mRNA (upper) and protein (lower) levels of FOLR1 in two pairs stable cell lines of (**B**) FUT8 overexpression or (**C**) knockdown. The cell lines with FUT8 knockdown were further treated by HGF for 48 h before their FOLR1 mRNA measurement. **D.** Immunofluorescence staining of FUT8 over-expressed three cell lines treated with 300 µM FITC-FA for 24 h. Scale bar, 30 µm. Relative intensity of FITC-labeled green immunofluorescent was determined by ImageJ software (n=10). **E.** Immunofluorescence staining of siFUT8-mediated knockdown three cell lines treated with FITC-FA for 24 h. Scale bar, 50 µm. Relative intensity of red and green immunofluorescent was determined by Image-J software (n=10). **F.** Effects of various concentrations of folate (100~1200 µM) on the protein levels of FUT8, N-cadherin and E-cadherin in SMMC-7721 and HepG2 cells. All qPCR data were generated by averaging triplicate analyses per condition. GAPDH was used as a control. Data are presented as mean ± SEM. P values were determined by two-tailed unpaired t-test in (B-D), or One-way ANOVA was used in (E). *n.s*, no significance; *P < 0.05, **P < 0.01, ***P < 0.001, ****P < 0.0001.

To further examine the effects of FUT8 overexpression and knockdown on FOLR1 expression and core-fucosylation of the attached *N*-glycans, we measured the mRNA and protein expressions of FOLR1 in HGF-treated SMMC-7721 and HepG2 cells by using qRT-PCR and quantitative proteomic approaches, respectively. The results showed that FOLR1 had no significant alteration at mRNA and protein levels in both HCC cell lines treated by HGF (**Figure 5A**). In addition, both overexpression (**Figure 5B**) and knockdown (**Figure 5C**) of FUT8 didn't affect the expression of FOLR1 at mRNA and protein levels, but positively affected the overall core-fucosylation of proteins (**Figure 4F**-**G**) as well as core-fucosylation on FOLR1 (**Figure 4H**).

Previous studies have shown that out of three functional folate receptors in human (FOLR1, FOLR2, and FOLR3), only FOLR1 exists on the apical surface of polarized epithelial cells, mediating mainly cellular uptake of folate by binding and releasing folate from outside to inside of cells 35. When incubating FUT8 over-expressed cell lines with 300 μM FITC-labeled folates (FITC-FA), we found that the amount of green fluorescence of folate was increased compared with that of control (**Figure 5D**). Conversely, the amount of green fluorescence of folate was significantly reduced by Cy3-labeled siFUT8 compared with that of control cells (**Figure 5E**). These results indicated that FUT8 can positively regulate uptake capacity of folate in HCC cells, possibly via the increased core-fucosylation on FOLR1.

Furthermore, we measured the effect of different concentrations of folate on the status of HCC cell lines. Different concentrations (100~1200 μM) of folate did not affect the mRNA (**Figure S12A**-**B**) and protein (**Figure 5F**) expression of FUT8 in SMMC-7721 and HepG2 cell lines. Folate increased the expression of N-cadherin and decreased the expression of E-cadherin both in a dose-dependent manner (**Figure 5F**), indicating that “super-physiological” dose of folate solely can directly promote the EMT of HCC cells.

### Glycosylation on Asn-201 of FOLR1 is most essential for uptake capacity of folate

**Figure 6:**
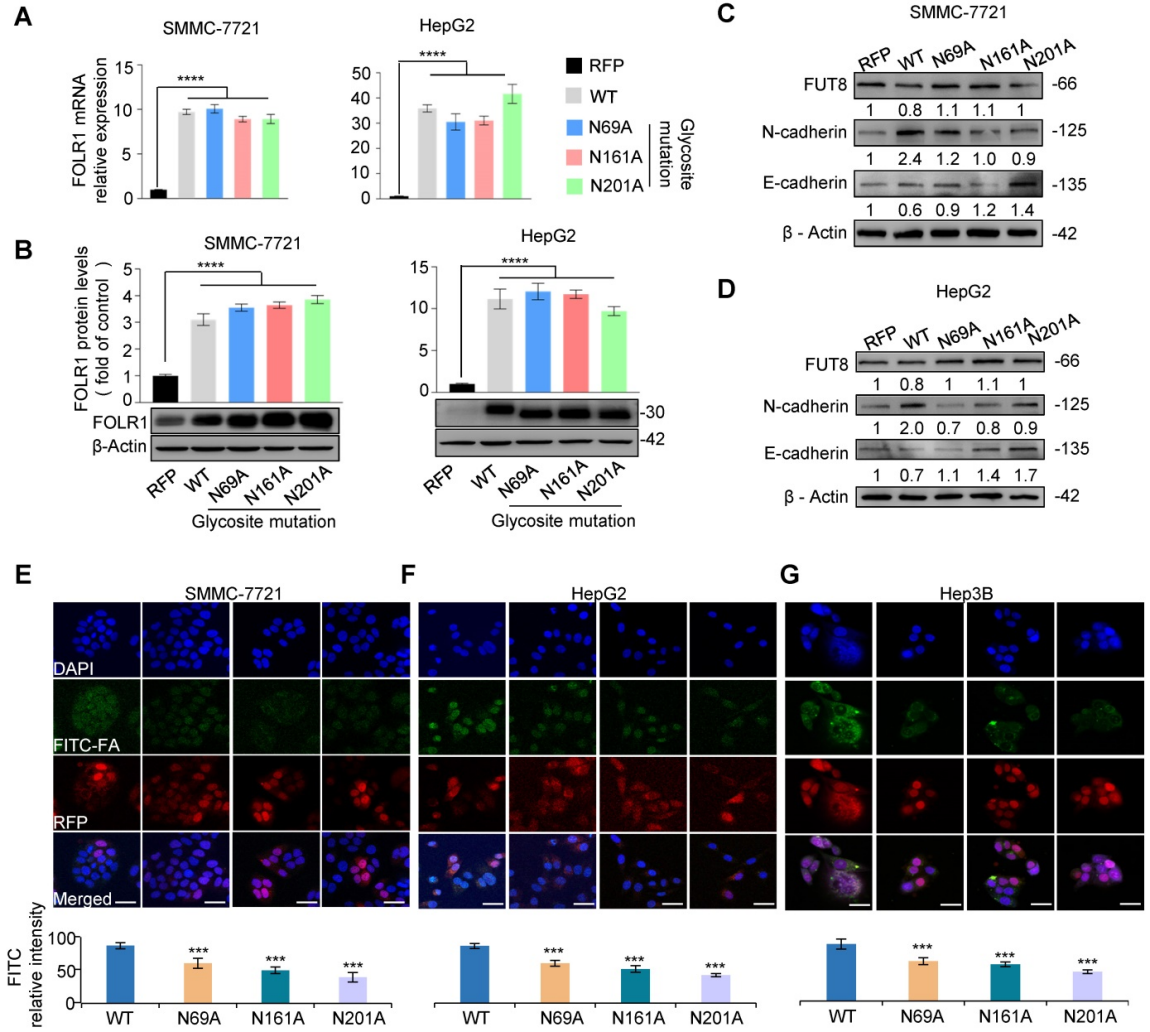
** Site-specific core fucosylation of FOLR1 is critical for folate uptake. A.** qRT-PCR analysis of FOLR1 mRNA expression level in five stable cell lines of site-specific glycosite mutations of FOLR1. The stable cell lines of RFP and FOLR1 wild-type (WT) were used as negative and positive controls, respectively. The data were generated by averaging at least triplicate analyses per condition. GAPDH was used as a control. **B.** The protein expression of FOLR1 in SMMC-7721 (left) and HepG2 (right) cell lines with glycosite mutations on FOLR1. **C, D.** The protein expression levels of FUT8, N-cadherin and E-cadherin in FOLR1 mutated cell lines treated by HGF (10 ng/mL) for 24 h. The grayscale value was measured from the western blotting data with Image J. **E-G.** Immunofluorescence staining of FOLR1 mutated cell lines incubated with FITC-FA for 24 h. 5 fields per replicate, 3 replicates per condition. Scale bar, 30 µm. Relative intensity of red and green immunofluorescent was determined by ImageJ software (n=10). Data are presented as mean ± SEM. P values were determined by One-way ANOVA. *n.s*, no significance, *P < 0.05, **P < 0.01, ***P < 0.001, ****P < 0.0001.

To further determine which glycosite of FOLR1 is critical for cellular uptake ability of folate, we mutated asparagine residues to alanine one by one at three *N*-glycosites on FOLR1, and constructed five stable cell lines of RFP, FOLR1 WT, FOLR1 N69A, N161A and N201A, respectively. The qPCR and western blot results showed that the mRNA (**Figure 6A**) and protein levels (**Figure 6B**) of FOLR1 were significantly increased in four stable cell lines including FOLR1 WT, N69A, N161A and N201A compared with the RFP, respectively. After treated with HGF for 24 h, the EMT progressions were partially blocked in all three glycosite-mutated cell lines in SMMC-7721 and HepG2 cells, according to the mRNA (**Figure S12C**-**D**) and protein (**Figure 6C-D**) alterations of E-cadherin and N-cadherin. Among three glycosites, the blocking effect of the mutation at the glycosite Asn-201 was the most significant.

To further evaluate effects of site-specific glycosite mutations of FOLR1 on uptake capability of folate, four stable cell lines were incubated with 300 μM FITC-FA. Compared with FOLR1 WT cells, the amount of green fluorescence from folate was significantly decreased in FOLR1 N69A, N161A and N201A cells (**Figure 6E**-**G**). These results suggested that the folate uptake capacity of FOLR1 was maximally affected by site-specific glycosylation of FOLR1, in particular the glycosylation of glycosite Asn-201. In addition, these results further confirmed that the folate uptake capacity of HCC cells could be enhanced by the core-fucosylation of FOLR1.

### A proposed novel pathway for HGF-induced EMT of HCC cells

**Figure 7:**
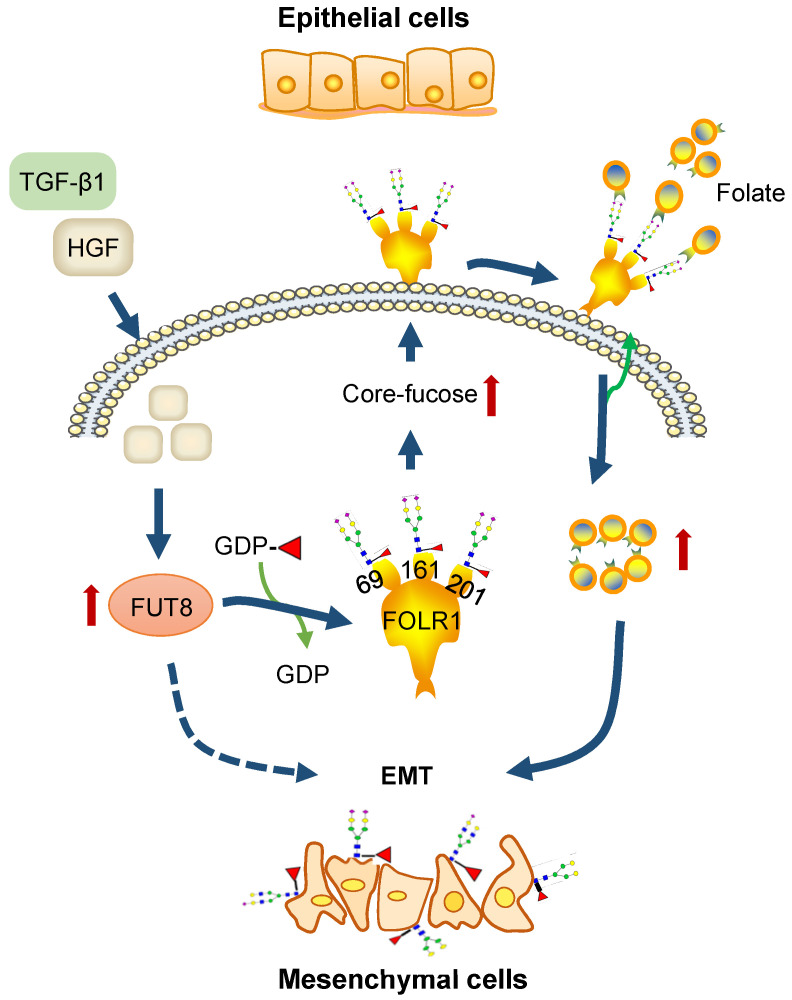
** A proposed novel pathway for HGF-induced EMT of HCC cells.** FUT8 is over-expressed in HGF or TGF-β1-treated HCC cells, which results in the up-regulation of core-fucosylation on FOLR1. The increased core-fucosylation on FOLR1 especially at the glycosite Asn-201 enhances the folate uptake capacity of the cell, providing sufficient dose of folates for promoting the EMT of HCC cells.

Based on above results, we concluded that HGF treatment of HCC cells can increase the expression of glycosyltransferase FUT8 to up-regulate the core-fucosylation of *N*-glycans on glycoproteins, especially FOLR1; core-fucosylation on FOLR1 can then enhance the folate uptake capacity of HCC cells to promote the progression of EMT (**Figure 7**). Within this pathway, our glycoproteomic data provided substantial evidence to reveal that the folate uptake capacity of FOLR1 can be positively regulated by site-specific core-fucosylation attached at FOLR1, which established a direct connection between the overexpression of FUT8 (and increased core-fucosylation) induced by HGF or TGF-β1 and the EMT of cancer cells stimulated by “super-physiological” dose of folate, which were only reported disjointly in previous studies 36-38.

## Discussion

EMT has been recognized as an important step toward the invasion and metastasis enhancement of HCC for decades. In previous studies, many alterations including the up-regulation of FUT8 39, increased core-fucosylation 40, excessive demands of folate, and the abnormal abundance of FOLR1 36-38 have been associated with tumor occurrence and development. It is also known that core-fucosylation is normally regulated by the expression level of FUT8, and folates were transported into the cells mainly by the cell surface folate receptor FOLR1. However, it seems that these two are independent stimulation methods for promoting the EMT of cancers. In this study, by systematically screening the dynamic changes of site-specific *N*-glycans in three HGF or TGF-β1 treated HCC cell lines, complemented by molecular biology approaches, we provided solid evidences to show that the folate uptake capacity of FOLR1 is enhanced by its increased site-specific core-fucosylation, and therefore links these two stimulation factors into one complete pathway 20.

Core-fucosylation, which was synthesized by the glycosyltransferase FUT8, has long been recognized as an important modification on N-linked glycans. It plays essential roles in modulating the affinity activity of many cell surface receptors with their ligands 41-46. The abnormity of core-fucosylation has been associated with many diseases, including congenital disorder of glycosylation 47, melanomas 39, and breast cancer 48. In recent studies, it has been reported that core-fucosylation is also elevated in liver tumors compared to normal liver tissues 20, and the up-regulation of FUT8 can promote HCC metastasis 49. The detailed functions and underlying mechanisms of core-fucosylation in the progression of HCC and HGF/TGF-β1-stimulated EMT, however, remain unknown. In this study, we also observed the increase of core-fucosylated glycans (and FUT8) in HGF/TGF-β1-treated HCC cells by using our site-specific glycoproteomics method. In addition, direct analysis of intact glycopeptides allowed us to further identify FOLR1 as an important target of elevated FUT8 for core-fucosylation during EMT, which is the key to link up-regulation of FUT8 with increased folate uptake for EMT promotion.

Among three known functional folate receptors, FOLR1 and FOLR2 are located at the plasma membrane of the human cells, while FOLR3 is a secreted protein 35, 50-54. FOLR1 is mainly displayed on the cell surface of epithelial cells, while FOLR2 is usually expressed in the latter stages of normal myelopoiesis and in the placenta, spleen, and thymus 55, 56. As an important cell-surface glycoprotein on epithelial cells, FOLR1 can bind folate with high affinity to mediate its cellular uptake for cell metabolism as well as DNA synthesis and repair 56. It has relatively high affinity for folate and folate analogs at neutral pH, and a lower affinity at slightly acidic pH after endocytosis to facilitate the release of folates. FOLR1 has restricted expression in normal tissues but is highly expressed in specific malignant tumor mainly to meet the folate demand of rapidly dividing cells 35-38, 55. It therefore has been used as an important target for cancer therapy 35, 50. In this study, we further confirmed that high concentration of folate can promote the EMT of HCC cells. In addition to providing excessive amounts of folate in the cell culture medium, we showed that the elevated folate uptake of the HCC cell could also be achieved by up-regulating the core-fucosylation at all glycosites of FOLR1, preferably at the glycosite Asn-201 (but not increasing the FOLR1 protein expression). In a previous study, Chen* et al* 55 reported that the de-glycosylated form of FOLR1 (only a single HexNAc moiety attached at each glycosylation site) had a similar folate-binding affinity with the fully glycosylated protein. This implies that the enhancement of folate uptake capacity by increased site-specific core-fucosylation on FOLR1 may mainly occur at the folate release and/or FOLR1 transportation steps.

## Conclusion

In conclusion, with site-specific glycoproteomics and molecular biology approaches, we demonstrated that HGF/TGF-β1 significantly up-regulated the expression of FUT8, which led to the increasing of core-fucosylation on FOLR1. This further increased the cellular uptake of folate and promoted the EMT of HCC cells. With glycosite mutations, we further identified Asn-201 as the most critical core-fucosylated glycosite for folate uptake and EMT progression. Based on these results, the core-fucosylation of FOLR1 especially at the glycosite Asn-201 may represent another promising marker and therapeutic target for HCC monitoring and treatment.

## Methods

### Cell culture and HGF/TGF-β1 treatment

Human HCC cell lines SMMC-7721, Hep3B and HepG2 were obtained from Cell Bank of the Chinese Academy of Sciences (Shanghai, China) and Tumor Cell Libraries of the Chinese Academy of Medical Sciences (Beijing, China), respectively. The cell lines were tested negative for mycoplasma contamination before our experiments. All cell lines were cultured in DMEM medium supplemented with 10% FBS and 1% penicillin-streptomycin at 37 °C in a humid atmosphere of 5% CO_2_ for the first three generations. Then the medium was changed to the basic medium (without FBS) for 8 h before HGF or TGF-β1 (10 ng/mL, Peprotech, USA) stimulation. HGF-treated cellular proteins were then harvested from three biological replicates at six different time points (only 0, 12 and 48h for Hep3B) within 72 h (0, 6, 12, 24, 48 and 72 h). TGF-β1-treated cellular proteins were harvested from three biological replicates at three different time point (0, 24 and 72 h). The proteins at each time point were pooled into one sample for further sample preparation.

### Protein extraction

Cells on the culturing dishes were washed three times with pre-cooled phosphate buffered saline (PBS, pH 7.4) and lysed directly with a lysis buffer with a final concentration of 8 M urea/1 M NH_4_HCO_3_ solution. High concentration of NH_4_HCO_3_ solution was used to inhibit the potential carbamylation of proteins and peptides in urea solution 58. Lysates were briefly sonicated until the solution was clear. The protein concentrations were measured by UV absorbances at 562 nm after reaction with BCA protein assay reagent (Beyotime, China) 59 using a Multiskan FC-3000 spectrophotometer (Thermo, USA).

### Protein digestion

The cellular proteins from different time points were first adjusted to the same concentration (~4 mg/mL). The proteins were then reduced by 5 mM dithiothreitol (DTT) at 37 °C for 1 h and alkylated by 15 mM iodoacetamide (IAM) at room temperature in the dark for 30 min. Another 2.5 mM DTT was then added and incubated at room temperature for 10 min. The proteins were diluted two times with deionized water and digested with sequencing grade trypsin (Promega, USA, WI; proteins: enzyme, 100:1, w/w) for 2 h with shaking at 37 °C. Furthermore, the proteins were digested for the second time with same amount of trypsin (proteins: enzyme, 100:1, w/w) overnight at 37 °C with shaking after another four-time dilution. A two-step digestion was mainly used to enhance the digestion efficiency of trypsin and thus reduce the overall missed cleavage rates of identified peptides. The pH of the solution was adjusted to < 2 with 10% TFA. The samples were centrifuged at 15,000 g for 10 min to remove any particulate matter. The peptides in supernatants were collected and desalted by HLB columns (Waters, USA). Peptides were eluted from HLB column in 60% ACN in 0.1% TFA solution. The peptide concentrations were measured by UV absorbance at 215 nm using a DS-11 spectrophotometer (Denovix, USA).

### TMT labeling

The tryptic peptides from different time points of HGF-treated cells were dried and re-dissolved in 50 mM HEPES solution (pH 8.5). Equal amounts of peptides (100 μg) from each time points were labeled by two sets of 10-plex Tandem Mass Tag (TMT, Thermo Fisher, USA) reagents according to the manufacturer's protocol. Set #1 was used to label cellular proteins from SMMC-7721 and Hep3B cell lines. TMT channels of 126, 127N, 127C, 128N, 128C, 129N were used to label HGF-treated cellular proteins from SMMC-7721 at 0, 6, 12, 24, 48 and 72 h, and 129C, 130C, 131 were used to label HGF-treated samples from Hep3B at 0, 12 and 48 h. Set #2 were used to label cellular proteins from HepG2 cell lines. TMT channels of 126, 127N, 127C, 128N, 128C, 129N were used to label HGF-treated samples at 0, 6, 12, 24, 48 and 72 h. The labeled samples within each TMT set were then pooled and desalted by HLB columns. About 20 μg labeled peptides were used for further proteomic analysis, and the remaining labeled peptides (~580 μg) were used for glycopeptide enrichment.

### Enrichment of intact glycopeptides using MAX columns

The intact glycopeptides were enriched from TMT labeled samples using an anion exchange reversed phase (MAX) column (Waters, USA) 22, 60. Briefly, the TMT labeled peptides eluted from HLB column were adjusted by 100% ACN and TFA to the final concentration of 95% ACN in 1% TFA. The MAX column was washed three times each by 100% ACN, 100 mM triethylammonium acetate buffer, deionized water, and 95% ACN in 1% TFA, followed by twice sample loading and four times washes with 95% ACN in 1% TFA. The glycopeptides bounding to the column were eluted in 400 μL of 50% ACN in 0.1% FA. The sample was dried by SpeedVac and resuspended in 20 μL of 0.1% FA.

### LC-MS/MS analysis

The peptides and enriched glycopeptides were analyzed by Orbitrap Fusion Lumos Mass Spectrometer (Thermo Fisher Scientific, Germany) in duplicates. One microgram of peptides were first separated on an EASY-nLC™ 1200 instrument (Thermo Fisher Scientific, Germany) with a 75 μm × 50 cm acclaim PepMap separating C18 column (Thermo Fisher Scientific, Germany) protected by a 75 μm × 2 cm guarding column. The mobile phase A: 0.1% formic acid in water and B: 0.1% formic acid in 80% ACN with a flow rate at 200 nL/min. The gradient profile (180 min) for labeled samples was set as follows: 3-7% B for 1 min, 7-25% B for 104 min, 25-35% B for 50 min, 35-70% B for 14 min, 70-100% B for 1 min and 100% B for 10 min. The gradient profile (240 min) for unlabeled samples was set as follows: 3-40% B for 203 min, 40-68% B for 20 min, 68-99% B for 4 min, 99% B for 13 min.

The mass spectrometry settings for proteomic analysis were set as follows: the spray voltage was set at 2.4 kV, MS1 spectra (AGC 4.0 × 10^5^) were collected from 350-1,800 m/z at a resolution of 60 K followed by data-dependent HCD tandem mass spectrometry (HCD-MS/MS) with the collision energy of 37%, RF lens at 60%, and intensity threshold of 2.0×10^4^. The charge states for peptides were 2-7. A dynamic exclusion time of 10 s was used to discriminate against previously selected ions for MS/MS fragmentation.

The settings for labeled intact glycopeptide analysis were set as follows: the spray voltage was set at 2.4 kV. Orbitrap MS1 spectra (AGC 4.0×10^5^) were collected from 500-1,800 m/z at a resolution of 60 K. Each selected MS1 peak (isolation width of 2 m/z) was fragmented by data-dependent HCD with collision energy of 20% and 37% at a resolution of 50 K (two MS2 spectra per glycopeptide/peptide). High HCD energy of 37% was used for identification of the peptide sequence, while low HCD energy of 20% was used for glycan structure analysis of the intact glycopeptide. The charge states for intact glycopeptide were set as 3-7. A dynamic exclusion time of 10 s was used to discriminate against previously selected ions.

The settings for unlabeled intact glycopeptide analysis were set as follows: the spray voltage was set at 2.4 kV. Orbitrap MS1 spectra (AGC 4.0×10^5^) were collected from 375-2,000 m/z at a resolution of 120 K. Each selected MS1 peak (isolation width of 2 m/z) was fragmented by data-dependent HCD with collision energy of 33% and 20% at a resolution of 30 K (two MS2 spectra per glycopeptide/peptide). High HCD energy of 33% was used for identification of the peptide sequence, while low HCD energy of 20% was used for glycan structure analysis of the intact glycopeptide. The charge states for intact glycopeptide were set as 2-7. A dynamic exclusion time of 20 s was used to discriminate against previously selected ions.

### Database search for protein identification and quantification

For global proteomics data, the MS/MS spectra were searched against reviewed human protein database from Uniprot website (www.uniprot.org, downloaded at May 15, 2017) by Thermo Proteome Discoverer 2.2 (Thermo Fisher Scientific, Germany). The search parameters were set as follows: up to two missed cleavage sites were permitted for trypsin digestion, 10 ppm precursor mass tolerance, 0.06 Da fragment mass tolerance, carbamidomethylation (C, +57.0215 Da) and TMT10plex (N-termini of peptides, +229.162932 Da) were set as fixed modifications, and oxidization (M, +15.9949 Da), TMT10plex (K, +229.162932 Da), and deamidation (N, +0.98 Da) were set as dynamic modifications. The results were filtered with 1% FDR and at least two peptide-spectrum matches (PSMs) were required for each peptide. The peptides were quantified based on the intensities of TMT reporter ions released from TMT-labeled peptides.

### Intact glycopeptide identification

The MS data of intact *N*-glycopeptides were first converted to 'mzML' format using Trans-Proteome Pipeline (TPP) 61 and searched by our in-house software StrucGP, a data-independent glycopeptide analysis software for structural interpretation of site-specific *N-*glycans (https://github.com/Sun-GlycoLab/StrucGP). As each intact glycopeptide was fragmented by two individual HCD energies (HCD = 37% and HCD = 20%) in one LC-MS/MS analysis, the sequence of the peptide portion was identified by matched b and y ions from high HCD energy of MS/MS spectrum (HCD = 37%), while the glycan structure was identified by using B and Y ions from low HCD energy of MS/MS spectra (HCD = 20%). The search parameters were set as follows: at least two oxonium ions out of the top 10 fragment ions in the MS/MS spectra were used for extraction of intact glycopeptide MS/MS spectra. The mass tolerances of 10 ppm and 20 ppm were allowed for precursor ions and fragmentation ions. The tryptic peptides with N-X-S/T motifs (X is any amino acid except proline) were used as potential glycosite-containing peptides for database search (with up to two missed cleavage sites). The identified intact glycopeptides were filtered by ≥4 matched b/y ions of the peptide portion in the MS/MS spectra. A target-decoy database method was used for peptide FDR evaluation, which has been described in GPQuest 2.0 62. In addition, a series of decoy MS/MS spectra were generated in silico by adding a random mass (ranging from 10-140 Da) to the precursor mass of each MS/MS spectra while keeping MS2 information unchanged, and these decoy MS/MS spectra were used to evaluate the FDR of glycan structures. The 1% FDR was used as the cutoff for the identification of both peptide sequences and glycan structures of intact glycopeptides.

### Intact glycopeptide TMT quantification

The intensities of their TMT reporter ions were extracted from the identified MS/MS spectra with high energy HCD fragmentation (HCD = 37%) for relative quantification of intact glycopeptides. Intact glycopeptides with PSM≥5 in both cell lines were used for further quantification analysis. The median ratios of given glycopeptides were used as quantification results, and the quantification results were further normalized by the normalization factors obtained from the global proteomic results.

### Intact glycopeptide label-free quantification

The Label-free quantification (LFQ) of intact N-glycopeptides were performed using Byonic 24 and Proteome Discoverer 2.2. The search parameters were set as follow: a maximum of two missed cleavage sites were allowed for trypsin digestion; the mass tolerances of 10 ppm and 20 ppm were allowed for precursor ions and fragmentation ions; carbamidomethylation (C, +57.0215 Da) was set as fixed modifications, and N-terminal acetylation (+21.011 Da) and oxidization (M, +15.9949 Da) were set as dynamic modifications. The results were filtered with 1% FDR. The quantification information of assigned glycopeptide spectra (based on their MS/MS scan numbers) were extracted from PSMs files from Byonic and Proteome Discoverer 2.2 results using StrucGP software. For each analyzed sample, intact glycopeptide intensities were normalized to the median peptide intensity of the respective data set to adjust for possible variation between analyses. The median of all pairwise ratios between replicates was calculated as the ratio of intact glycopeptide.

### Gene ontology and KEGG pathway analyses

Gene Ontology (GO) enrichment analysis of differentially expressed proteins in both cell lines were performed using David Bioinformatics tools (https://david.ncifcrf.gov/). The thresholds of >1.5-fold changes, < 0.67-fold changes, and count >2 were applied as filters on GO analysis of altered proteins. The subset of proteins identified in this study were used as background for GO analysis. STRING was used to analyze functional protein interaction networks 63.

### Folate treatment

SMMC-7721 and HepG2 cell lines were planted into 6-well plates and cultured overnight. Different concentrations of folate (100, 300, 600, 900, 1200 μM) (Sigma, USA) were added into the cells and incubated for 24 h. Finally, folate-treated cells were harvested and prepared for qRT-PCR and western blot analysis.

### Cell transfection

Three cell lines (~1×10^6^) were seeded into 6-well plate and incubated overnight. Then, cells were transfected with 100 nM Cy3-labeled siFUT8 (Ribobio, China) and Cy3-labeled siScramble (Ribobio, China) using Lipo8000™ transfection reagent (Beyotime, China) following manufacturer's recommendations. All the sequences were available in Supplementary **Table S7**.

### Plasmid constructs

The overexpression plasmid was constructed using pCDH-CMV-MCS-EF1-Puro vector inserted with the coding sequence of human FUT8. FUT8 knockdown plasmid was constructed using pGreen-Puro vector with insertion of short-hairpin(sh) RNAs targeting FUT8 (GeneCreate, China). The targeted sequences of FUT8 and scramble siRNA were available in **Table S7**. FOLR1 contains three known *N*-glycosylation sites including Asn69, Asn161 and Asn201. The asparagine residues at three glycosites were mutated to alanine one by one. The wild-type (WT) and mutants (N69A, N161A and N201A) of FOLR1 were inserted into the pCDH-EF-1a-MCS-RFP-Puro vector for stable cell line establishment. All plasmids were sequenced for verification (BGI, China).

### Lentivirus production

HEK293T cells (~2.0×10^6^) were seeded into 10 cm dish and incubated for 24 h. Cells were co-transfected with 3 μg expression vector, 2.5 μg viral packaging plasmid pSBH155 and 2.5 μg viral envelope plasmid pSBH156 using PEI reagent (Polysciences Inc, USA) following manufacturer's recommendations. After transfection for 48 h, cell supernatant was collected and filtered with 0.45 μm filter (Merck, USA). Virus supernatant was subpackaged and stored at -80 °C.

### Lentiviral transduction and stable cell lines

SMMC-7721, Hep3B and HepG2 were cultured overnight prior to infection. Three cell lines were infected with 1:3 diluted viral supernatant for 24 h, and the medium was then replaced with DMEM supplemented with 10% FBS. After incubation for 48 h, each cell line was selected with 2 μg/mL puromycin prior to use in experiments.

### Cell invasion assay

Transwell Permeable Supports with 24 wells (insert diameter: 6.5 mm; aperture: 8.0 μm) (Corning, USA) and growth factor-reduced Matrigel (R&D, USA) were used for cell invasion assay^39^. The stable cells with FUT8 overexpression (~1×10^5^ clones) or knockdown (~1×10^4^ clones) in serum-free medium were added to the upper chamber. Medium supplemented with 10% FBS was added to the lower chamber as a chemoattractant. Cells were then incubated at 37 °C in a humid atmosphere of 5% CO_2_ for 24 h. For invaded cells with FUT8 overexpression, the cells were fixed with 4% formaldehyde for 15 min, and then stained by crystal violet. For invaded cells with FUT8 knockdown, the cells were fixed with 4% formaldehyde for 15 min. Different fields of green fluorescent cells were then randomly selected and photographed. The average of cell counts in five microscope fields per condition was used for plotting results.

### Immunofluorescence staining

SMMC-7721, Hep3B and HepG2 (5×10^3^ cells) were seeded on glass coverslips and incubated overnight. Then cells were transfected with 100 nM Cy3-siFUT8 or Cy3-siScramble (Ribobio, China) using Lipo8000 reagent (Beyotime, China). Then, cells were incubated with 300 μM FITC-FA (Sigma, USA) for another 24 h after 24 h of transfection. After fixed with 4% paraformaldehyde for 15 min at room temperature, the cells were washed with cold PBS buffer and stained with 5 μg/mL DAPI (Beyotime, China) for 5 min at room temperature. After an additional wash with PBS, anti-fade reagent was applied onto glass slides. Immunofluorescence images were acquired by the fluorescence microscopy (ZEISS, Germany). Relative intensity of immunofluorescence was quantified by Image-J software.

### RNA isolation and real time PCR

Total RNA was isolated using TRIZOL reagents according to the manufacturer's protocol (Life Technologies, USA). Half microgram of total RNA was prepared through reverse transcription using the PrimeScript TM II 1st strand cDNA synthesis kit (TaKaRa, Japan). qRT-PCR was performed with SYBR Green PCR Master Mix (TaKaRa, Japan) according to the manufacturer's protocol. The primers of homo FUT8, FOLR1, E-cadherin, N-cadherin, and GAPDH were listed in **Table S7**. All measurements were performed in triplicates. The mRNA level of GAPDH was used to normalize the relative expression of target mRNAs among different samples. The data were analyzed using the 2^-ΔΔCt^ method 64.

### Protein identification and label-free quantification

The steps for sample preparation and LC-MS/MS analysis of peptides from control, FUT8 OE, shControl and shFUT8 stable cell lines were the same as described above. The LC-MS/MS data were searched against the same human protein database by Proteome Discoverer 2.2. The search parameters were set as follows: a maximum of two missed cleavage sites permitted for trypsin digestion, 10 ppm precursor mass tolerance, 0.02 Da fragment mass tolerance, carbamidomethylation (C, +57.0215 Da) as a fixed modification, N-terminal acetylation (+21.011 Da) and oxidization (M, +15.9949 Da) as dynamic modifications. The results were filtered with 1% FDR and at least two PSMs were required for a peptide. LFQ was performed for quantification of identified peptides and protein groups using Proteome Discoverer 2.2. For each sample, total intensities of identified peptides were used to normalize peptide changes between LC-MS/MS runs.

### Western blot

HCC cell lines were lysed with cold RIPA buffer. The total proteins were extracted and separated on 10% SDS-PAGE gels, and transferred to 0.45 μm PVDF membranes (Millipore, USA). Membranes were blocked with 5% non-fat milk at room temperature for 1 h followed by incubation with primary and secondary antibodies. The following antibodies were used: anti-FUT8, anti-N-cadherin and anti-FOLR1 (Abcam, UK), anti-E-cadherin (Cell Signaling Technology, USA). Relative intensities of bands on the membranes were quantified by Image-J software.

### Statistical analysis

Data were analyzed with GraphPad Prism 7.0 software (La Jolla, CA, USA). All values were presented as mean ± SEM. All data was analyzed by two-tailed unpaired t-test (two groups) or one-way ANOVA followed by Tukey post-hoc test (three groups or more). MultiExperiment Viewer (Mev) 65 was used for hierarchical clustering of altered proteins and intact glycopeptides, and the distances among clusters were calculated based on Euclidean distance.
