# Supplementary material for: Site-specific glycoproteomic analysis revealing increased core-fucosylation on FOLR1 enhances folate uptake capacity of HCC cells to promote EMT
Source: Theranostics. 2021 May 8;11(14):6905–21. doi: 10.7150/thno.56882 (PMC8171077; doi:10.7150/thno.56882)
Supplement: Supplementary file 1 — Supplementary figures. [file thnov11p6905s1.pdf]

Supporting information for

Site-specific glycoproteomic analysis revealing increased core-fucosylation on FOLR1 enhances folate uptake capacity of HCC cells to promote EMT

Li Jia[1], Jun Li[1], Pengfei Li[1], Didi Liu[1], Jing Li[1], Jiechen Shen[1], Bojing Zhu[1], Chen Ma[1], Ting Zhao[1], Rongxia Lan[1], Liuyi Dang[1], Wang Li[2], Shisheng Sun[1]*

[1]College of Life Science, Northwest University, Xi'an, Shaanxi province 710069, China.

[2]Key Laboratory of Regenerative Biology, Guangzhou Institutes of Biomedicine and Health, Chinese Academy of Sciences, Guangzhou province 510530, China.

*Correspondence should be addressed to Shisheng Sun. (suns@nwu.edu.cn)

Supplementary Figures and Tables:

Figure S1 – S12 Page S3 – S14

Table S1. Intact glycopeptides identified from SMMC-7721 and HepG2 cell lines.

Table S2. Proteins identified from SMMC-7721 (A) and HepG2 (B) cell lines using mass spectrometry-based proteomics.

Table S3. Intact glycopeptides that were quantified among six time points of HGF-treatment in SMCC-7721 (A), HepG2 (B), and both cell lines (C) based on the TMT-labeled quantification method.

Table S4. Intact glycopeptides increased more than two-fold at I and/or M stages in both SMMC-7721 and HepG2 cell lines.

Table S5. Intact glycopeptides identified from FOLR1 in three cell lines.

Table S5. Intact glycopeptides identified from FOLR1 in three TGF-β1-treated HCC cell lines.

Table S7. Gene Primer sequences of homo FUT8, FOLR1, E-cadherin, N-cadherin, and GAPDH used for real-time PCR.

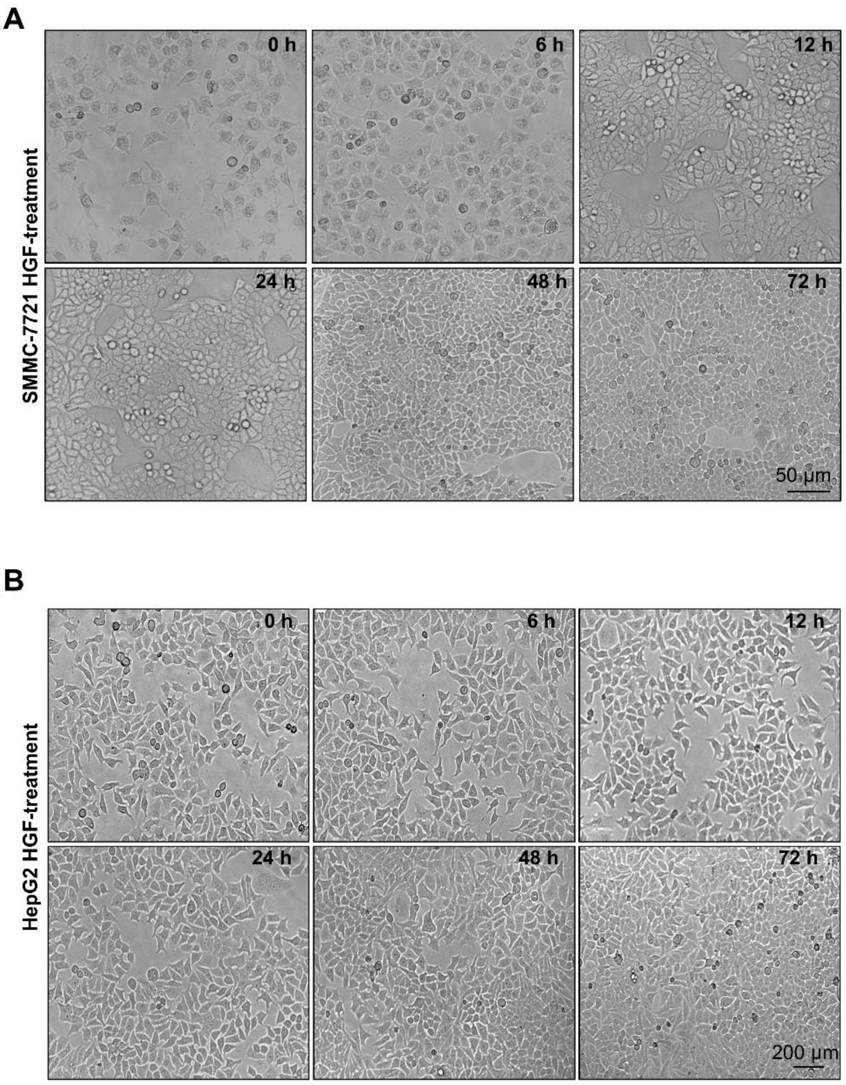

Figure S1. The morphological characteristics of SMMC-7721 (**A**, bar = 50 μm) and HepG2 (**B**, bar = 200 μm) with epithelial-mesenchymal transition induced by HGF. Data shown is the representative of three independent experiments. The numbers of treated cells increased from 30% to 90% coverage within three days in FBS free medium, a condition that the untreated cells could not survive.

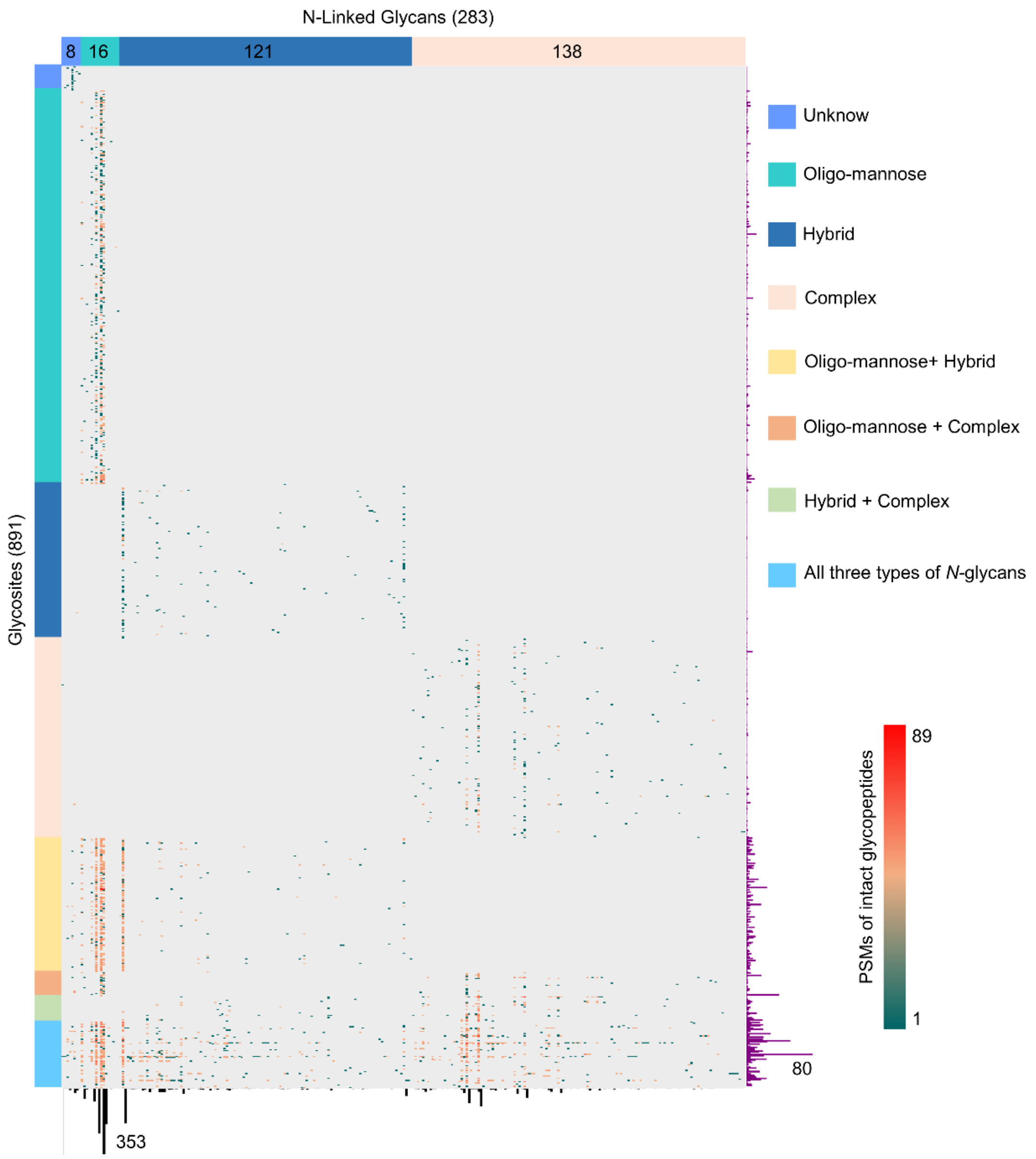

Figure S2. Heat map showing the intact glycopeptides identified from both SMMC-7721 and HepG2 cell lines. The peptide-spectrum-matches (PSMs) of each out of 848 intact glycopeptides, comprising of 283 glycan structures (159 compositions, upper) and 891 glycosites (left), were exhibited in the heat map with different colors. The histogram indicates the number of glycosites modified by each glycan (bottom) and glycans at each glycosite (right). There were up to 80 glycans with distinct structures were attached at a single glycosylation sites.

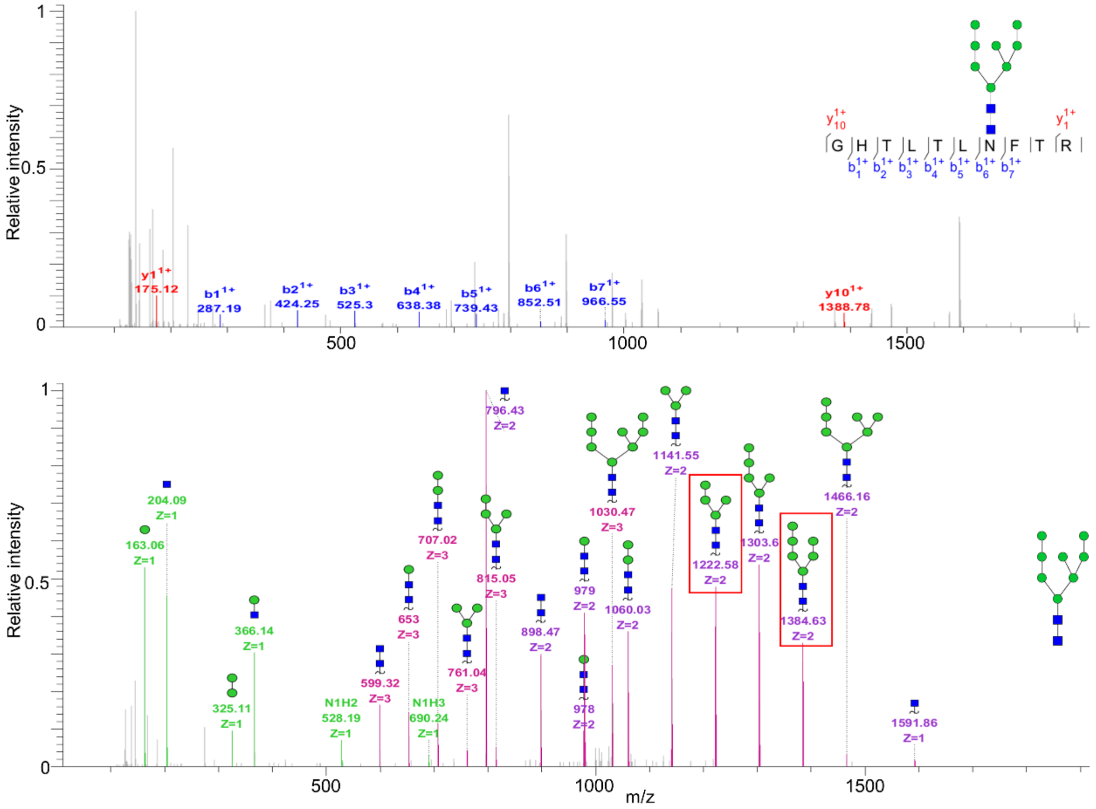

Figure S3. Representative MS/MS spectra for identification of an intact glycopeptide with peptide GHTLTL#NFTR modified by a high mannose glycan HexNAc2Hex8 (N2H8) from lysosome-associated membrane glycoprotein 1 (LAMP1). #indicates the glycosylation site. The intact glycopeptide was fragmented by two individual HCD energies (HCD=37% and HCD=20%) in a single LC-MS/MS analysis. The sequence of the peptide was identified by matched b and y ions (labeled in blue and red, respectively) from high HCD energy of MS/MS spectrum (HCD=37%, upper), and the glycan structure was determined by B and Y ions (labeled in green and purple, respectively) from low HCD energy of MS/MS spectra (HCD=20%, bottom). The glycan structure was identified using our in-house software StrucGP, and the labeled spectra were displayed using StrucGP Viewer. The feature Y ions for glycan subtype determination have been highlighted with red box.

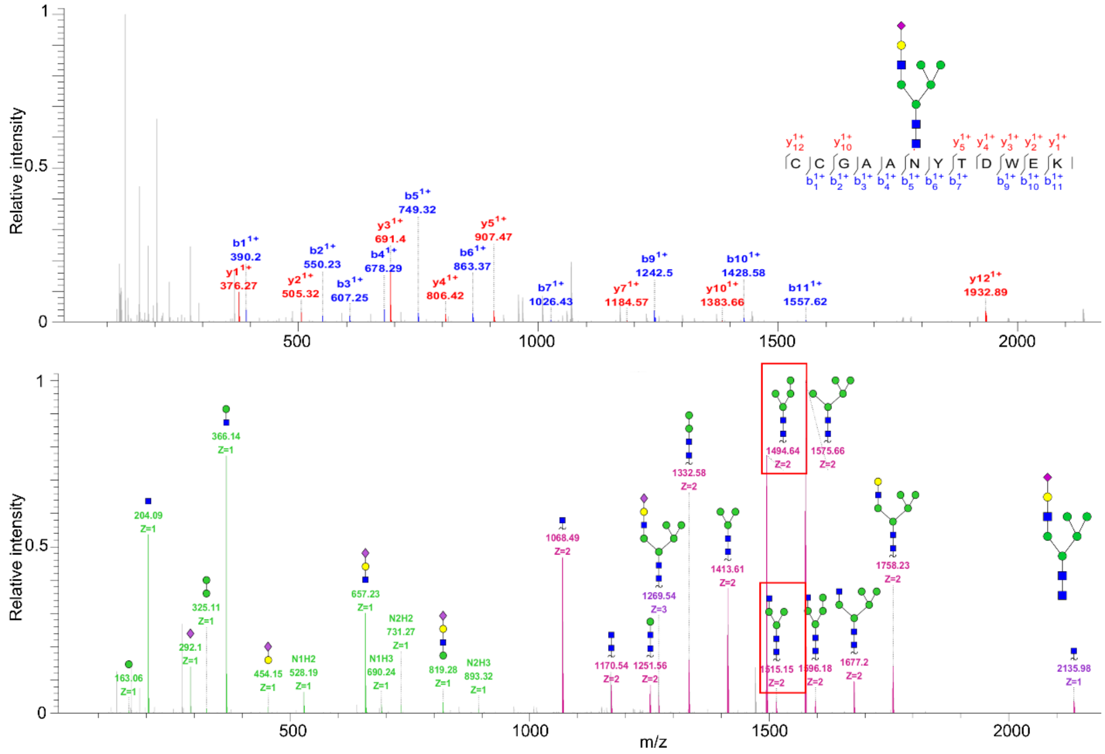

Figure S4. Representative MS/MS spectra for identification of an intact glycopeptide with peptide CCGAA#NYTDWEK modified by a hybrid glycan HexNAc3Hex6NeuAc1 (N3H6S1) from CD63 antigen (CD63). #indicates the glycosylation site. The intact glycopeptide was fragmented by two individual HCD energies (HCD=37% and HCD=20%) in a single LC-MS/MS analysis. The sequence of the peptide was identified by matched b and y ions (labeled in blue and red, respectively) from high HCD energy of MS/MS spectrum (HCD=37%, upper). The glycan was identified by B and Y ions (labeled in green and purple, respectively) from low HCD energy of MS/MS spectra (HCD=20%, bottom). The glycan structure was identified using our in-house software StrucGP, and the labeled spectra were displayed using StrucGP Viewer. The feature Y ions for glycan subtype determination have been highlighted with red box.

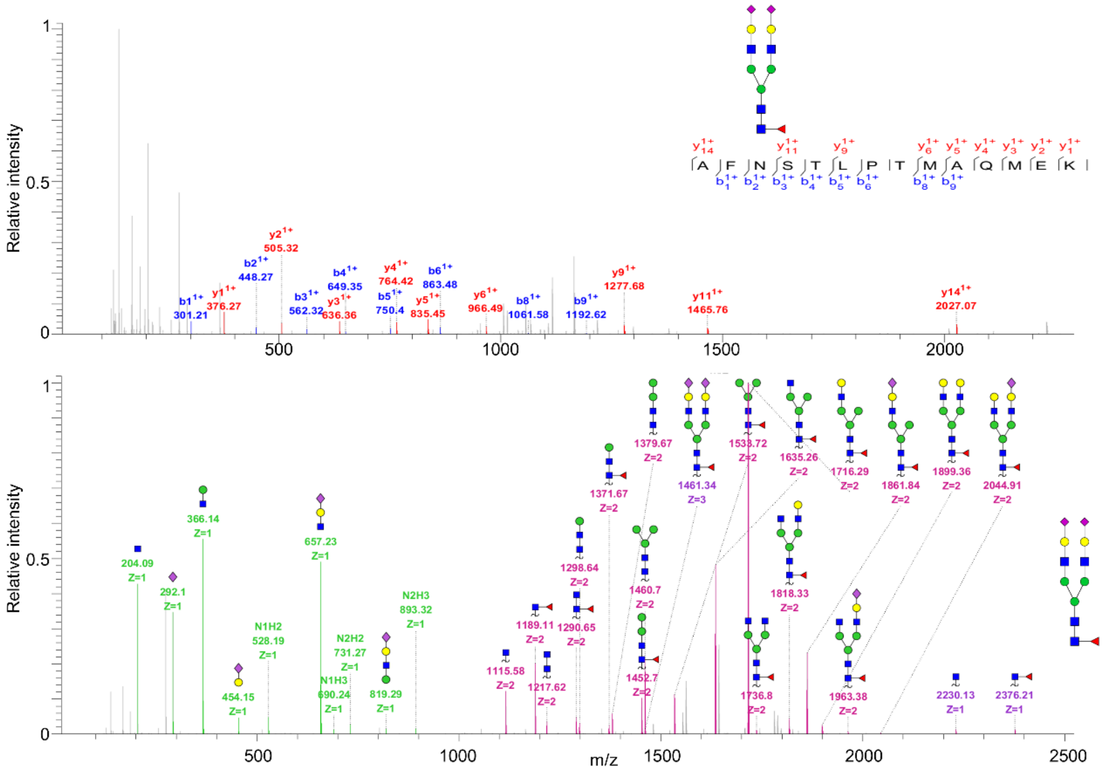

Figure S5. Representative MS/MS spectra for identification of an intact glycopeptide with peptide AF#NSTLPTMAQMEK modified by a complex glycan HexNAc4Hex5NeuAc2Fuc1 (N4H5S2F1) from CD44 antigen (CD44). #indicates the glycosylation site. The intact glycopeptide was fragmented by two individual HCD energies (HCD=37% and HCD=20%) in a single LC-MS/MS analysis. The sequence of the peptide was identified by matched b and y ions (labeled in blue and red, respectively) from high HCD energy of MS/MS spectrum (HCD=37%, upper). The glycan was identified by B and Y ions (labeled in green and purple, respectively) from low HCD energy of MS/MS spectra (HCD=20%, bottom). The glycan structure was identified using our in-house software StrucGP, and the labeled spectra were displayed using StrucGP Viewer. The feature Y ions for glycan subtype determination have been highlighted with red box.

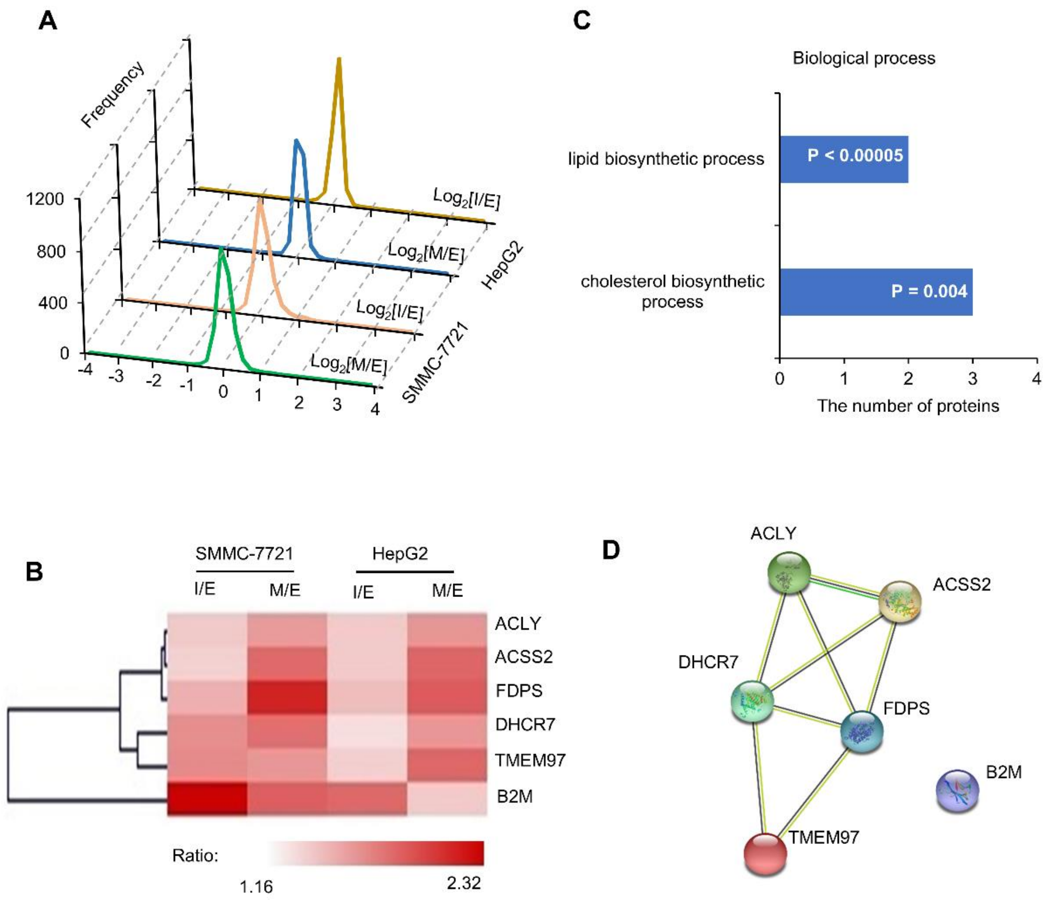

Figure S6. Quantitative proteome analysis of SMMC-7721 and HepG2 cell lines during the Epithelial-mesenchymal transition induced by HGF. **A.** Distribution of the protein alteration ratios at the intermediate (I) and mesenchymal (M) stages of EMT in both SMMC-7721 and HepG2 cell lines. *X axis*: $\log_2$(I/E) or $\log_2$(M/E); *Y axis:* the numbers of proteins. **B.** Hierarchical clustering of proteins that were changed more than two-fold at I and/or M stages in both cell lines. **C.** Biological processes involved by up-regulated proteins during the EMT process of both cell lines. **D.** Interaction of up-regulated proteins in the intermediate or mesenchymal stage of both cell lines. The interaction analysis was performed by using STRING.

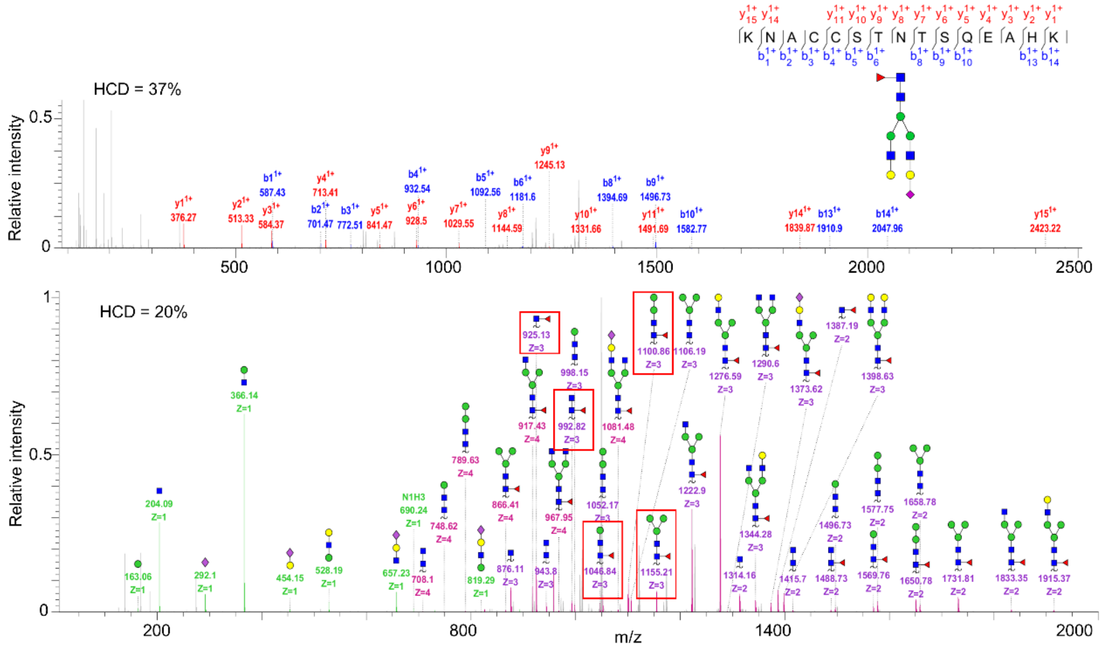

Figure S7. Representative MS/MS spectra for identification of an intact glycopeptide with peptide KNACCST#N201TSQEAHK modified by a complex glycan HexNAc4Hex5NeuAc1Fuc1 (N4H5S1F1) from FOLR1. #indicates the glycosylation site. The intact glycopeptide was fragmented by two individual HCD energies (HCD=37% and HCD=20%) in a single LC-MS/MS analysis. The sequence of the peptide was identified by matched b and y ions (labeled in blue and red, respectively) from high HCD energy of MS/MS spectrum (HCD=37%, upper). The glycan was identified by B and Y ions (labeled in green and purple, respectively) from low HCD energy of MS/MS spectra (HCD=20%, bottom). The glycan structure was identified using our in-house software StrucGP, and the labeled spectra were displayed using StrucGP Viewer. The feature Y ions for core-fucosylation determination are highlighted with red box.

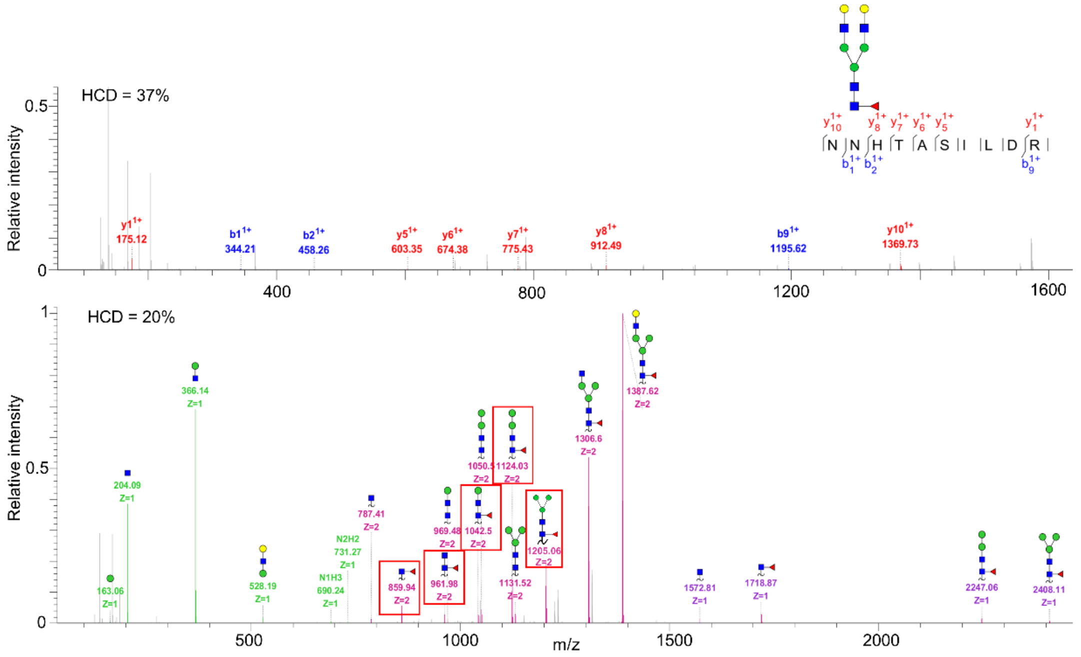

Figure S8. Representative MS/MS spectra for identification of an intact glycopeptide with peptide N#N130HTASILDR modified by a complex glycan HexNAc4Hex5Fuc1 (N4H5F1) from CD63 antigen (CD63). #indicates the glycosylation site. The intact glycopeptide was fragmented by two individual HCD energies (HCD=37% and HCD=20%) in a single LC-MS/MS analysis. The sequence of the peptide was identified by matched b and y ions (labeled in blue and red, respectively) from high HCD energy of MS/MS spectrum (HCD=37%, upper). The glycan was identified by B and Y ions (labeled in green and purple, respectively) from low HCD energy of MS/MS spectra (HCD=20%, bottom). The glycan structure was identified using our in-house software StrucGP, and the labeled spectra were displayed using StrucGP Viewer. The feature Y ions for core-fucosylation determination are highlighted with red box.

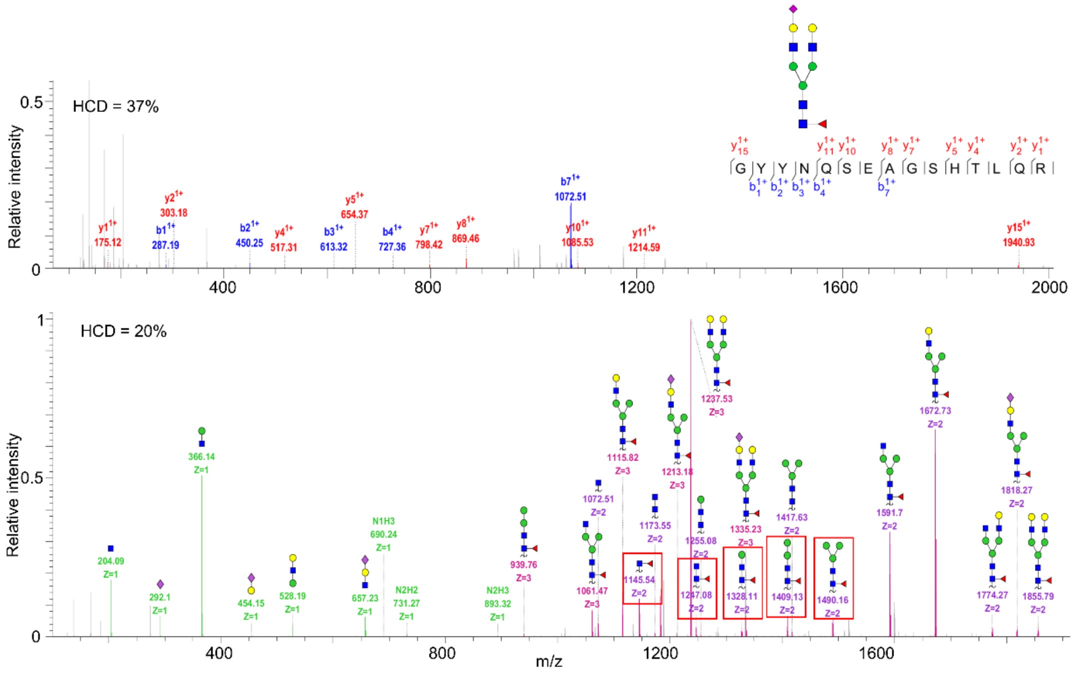

Figure S9. Representative MS/MS spectra for identification of an intact glycopeptide with peptide GYY#N^{110}QSEAGSHTLQR modified by a complex glycan HexNAc4Hex5NeuAc1Fuc1 (N4H5S1F1) from HLA class I histocompatibility antigen**,** C alpha chain (HLA-C). #indicates the glycosylation site. The intact glycopeptide was fragmented by two individual HCD energies (HCD=37% and HCD=20%) in a single LC-MS/MS analysis. The sequence of the peptide was identified by matched b and y ions (labeled in blue and red, respectively) from high HCD energy of MS/MS spectrum (HCD=37%, upper). The glycan was identified by B and Y ions (labeled in green and purple, respectively) from low HCD energy of MS/MS spectra (HCD=20%, bottom). The glycan structure was identified using our in-house software StrucGP, and the labeled spectra were displayed using StrucGP Viewer. The feature Y ions for core-fucosylation determination are highlighted with red box.

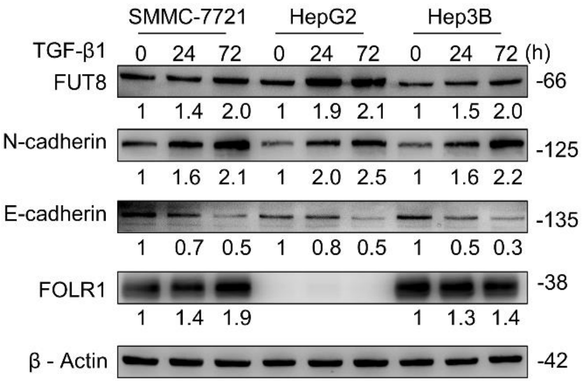

Figure S10. Measurement of protein expression in TGF-β1 treated HCC cell lines. N-cadherin and E-cadherin were used to evaluate the EMT process of three HCC cell lines. The grayscale values of each molecule was measured from the western blotting data with Image-J, and normalized based on the grayscale values of β-Actin.

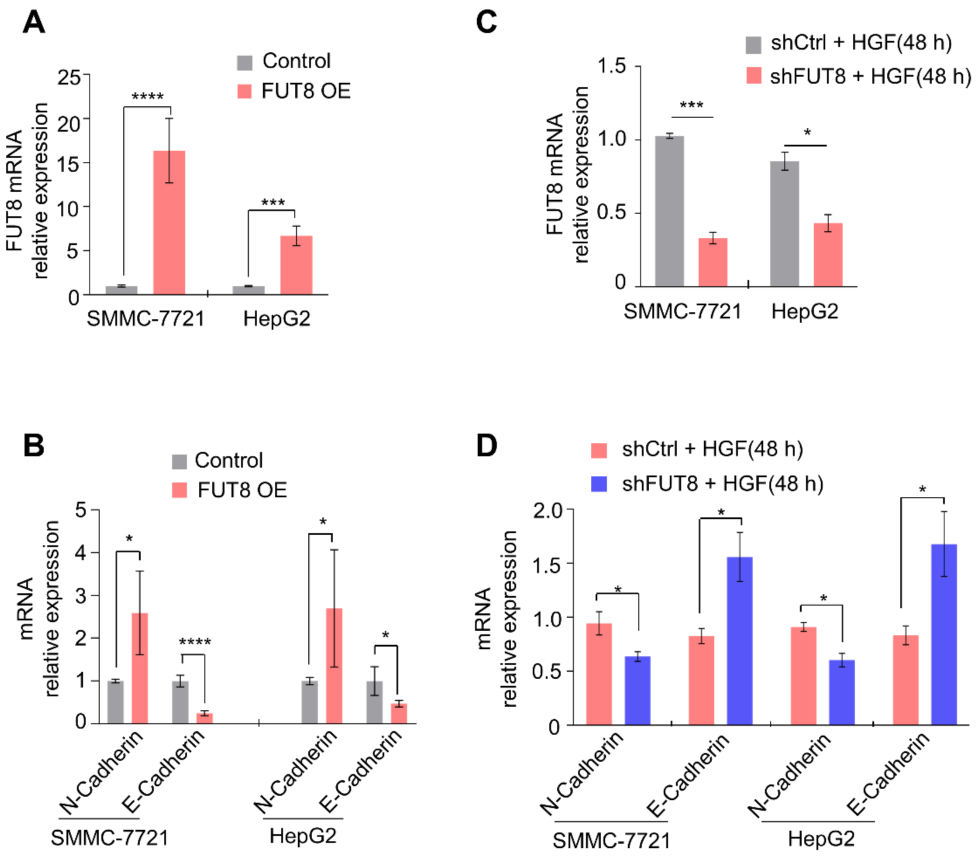

Figure S11. FUT8 promoted EMT process of both HCC cell lines. **A, C.** The mRNA levels of FUT8 in stable cell lines with FUT8 overexpression (**A**) and knockdown (**C**). **B, D.** The mRNA expressions of N-cadherin and E-cadherin in stable HCC cell lines with FUT8 overexpression (**B**) and knockdown (**D**). All data were generated by averaging triplicate analyses per condition. GAPDH was used as a control. Data are presented as mean±SEM; P-values were determined by unpaired two-tailed t-test. *P < 0.05, **P < 0.01, ***P < 0.001, ****P < 0.0001.

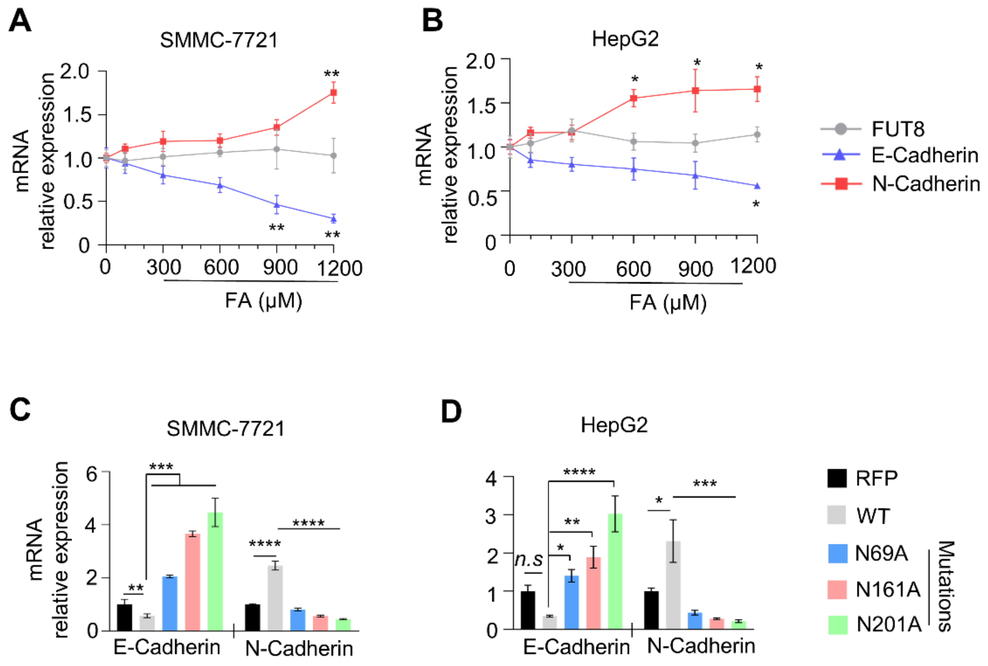

Figure S12. Folate promoted the EMT process of SMMC-7721 and HepG2 cells. **A, B.** Effects of various concentrations of folate (100~1200 μM) on the mRNA levels of FUT8, N-cadherin and E-cadherin in (**A**) SMMC-7721 and (**B**) HepG2 cells. **C, D.** The mRNA expression levels of N-cadherin and E-cadherin in FOLR1 mutated cell lines treated by HGF (10 ng/mL) for 24 h. The data were generated by averaging at least triplicate analyses per condition. GAPDH was used as a control. Data are presented as mean ± SEM. P values were determined by One-way ANOVA. n.s, no significance, *P < 0.05, **P < 0.01, ***P < 0.001, ****P < 0.0001.
